# Effects of the Application of *Pseudomonas cedrina* DY1-3 on the Growth of Maize Plants and the Structure of Soil Bacterial Community

**DOI:** 10.3390/microorganisms12122556

**Published:** 2024-12-11

**Authors:** Zhenzhen Liu, Yanlei Shi, Ye Yuan, Yonghong Fan, Peng Chen, Yingying Feng, Mengkedala Ningjing, Haocheng Li, Daiping Li, Lewei Wu

**Affiliations:** National Demonstration Center for Experimental Biology Education, Xinjiang Key Laboratory of Biological Resources and Genetic Engineering, College of Life Science & Technology, Xinjiang University, Urumqi 830017, China; lzz@stu.xju.edu.cn (Z.L.); stone_971107@163.com (Y.S.); yeyuanwilde@foxmail.com (Y.Y.); 13573373064@163.com (P.C.); 107552201029@stu.xju.edu.cn (Y.F.); 20211106806@stu.xju.edu.cn (M.N.); 20181106215@stu.xju.edu.cn (H.L.); l2107347894@126.com (D.L.); 15299500394@163.com (L.W.)

**Keywords:** plant growth-promoting bacteria, sequencing of soil bacterial 16S rRNA V3-V4 region amplicons, soil physico-chemical properties, saline soil improvement

## Abstract

Against the background of increasing salinisation of land, the use of environmentally friendly plant growth-promoting bacteria (PGPB) resources for soil improvement is particularly important. The aim of this study was to investigate the effects of *Pseudomonas cedrina* DY1-3 on maize seedling growth, soil physico-chemical properties, and bacterial community structure. The study also evaluates the effects of this microbial agent on plant growth and saline soil improvement, providing theoretical references for microbial agents in promoting plant growth and improving saline soils. We found that there were significant differences between arable and saline soils in terms of soil physico-chemical properties and bacterial community structural composition, and that total salt was the main environmental factor influencing microbial communities. In both arable and saline soils, the application of DY1-3 bacterial suspension had a significant positive effect on the growth of maize plants and bacterial community richness. In arable soil, it could promote the growth of maize seedlings and significantly increase the Shannon and Simpson index, and AK was a key factor influencing the bacterial community. In saline soil, it could alleviate the mitigation stress and promote the growth of maize seedlings and cause a significant increase in Shannon’s and Chao1 index, and the application of DY1-3 and potting could cause a significant decrease in total salt. In addition, DY1-3 and maize plants acting together in the soil can better improve the saline soil. The above results indicate that DY1-3 has potential for saline soil improvement and crop yield enhancement.

## 1. Introduction

Soil salinisation is the accumulation of soluble salts in the top layer of the soil due to natural factors such as specific climatic and geographic environments, as well as human factors such as irrational land-use practices [1]. In recent years, with the effects of global climate change and human activities, soil salinisation has been increasing, and the area of land affected by salinisation has been increasing, becoming a key constraint to agricultural production [2,3]. It was found that the inhibition of rice yield varied with different levels of saline stress, as evidenced by a high inhibition of rice yield ranging from 81.0% to 93.7% under severe saline stress, from 10.4% to 81.3% under moderate saline stress, and from 5.6% to 77.9% under mild saline stress [4]. In order to increase the resistance of ecosystems to soil salinisation, different preventive and ameliorative measures have been proactively adopted in salinisation-affected areas in various countries [5]. At present, there are three main methods to improve saline–alkaline land: physical, chemical, and biological, among which the physical and chemical methods have poor prevention and control effects, short duration, and ignore the integrity of the ecosystem, which is easy to cause ‘secondary pollution’ [6,7,8,9]. Therefore, there is an urgent need to develop cost-effective, efficient, and environmentally friendly strategies to address these problems. The bio-amended remediation methods are environmentally friendly and highly sustainable, thus making microbial agents a better choice for soil salinisation management [10,11,12,13].

Some soil microorganisms can play an important role in improving the soil environment, promoting plant growth and development, and enhancing plant resistance, and such microorganisms are known as PGPB [14,15,16]. It has been found that plant inter-root biotrophic bacteria can exert beneficial effects on soil microbial community structure and soil physico-chemical properties, plant growth, and abiotic stress response through a variety of mechanisms of action, which in turn improves the undesirable traits of salinised soils, promotes nutrient cycling in soils, increases soil fertility, and regulates the structure and composition of soil microbial communities [17,18,19,20]. For example, Wu Yude et al. found that inter-root biotrophic bacteria can increase soil productivity and reduce the harm of salt stress on agricultural plants, which is a major way to help plants cope with salt stress; Yuan Bingqiang et al. found that the combination of inter-root biotrophic bacteria and biochar in salinised soils planted with tomato could increase the content of soil organic matter, total nutrients and quick-acting nutrients; and Wu Zhuhua et al. found that inoculation with the composite bacterial agent NJ2D+BJ04 improved the soil microenvironment and led to a significant increase in the abundance of species of dominant phylums of bacteria in the soil [21,22,23]. The community structure of soil microorganisms is closely related to the soil environment, and exploring the relationship between soil microbial diversity, community structure and species composition, and soil physico-chemical factors can help to elucidate the relationship between microbial community diversity and soil ecosystem functioning from the perspective of agricultural production [24,25]. Relevant studies have found that there is an obvious correlation between soil physicochemical factors and microorganisms; for example, the content of soil organic carbon and nitrate nitrogen will be significantly increased under fertilisation conditions, which are the dominant factors affecting the structure of soil microbial communities [26,27]. Most of the above studies focus on exploring the effects of bacterial strains on plants or soil in a single direction, and few scholars have carried out systematic studies on the relationship between soil, microorganisms, and plants, and the elucidation of the interactions among the three has an important role to play in guiding the increase in agricultural yields and soil improvement, as well as in guiding the sustainable development of practical agriculture [28,29].

Therefore, based on the reality of soil salinisation in arid zones, the present study will investigate the effects of the application of *Pseudomonas cedrina* DY1-3, an PGPB which was isolated from Tianshan No. 1 glacier in the laboratory in a special habitat and with several biotrophic properties such as ACC deaminase activity and iron-producing carrier [30,31], on the growth of maize plants and on the bacterial community structure and physicochemical factors of different types of soils, and evaluate the potential of DY1-3 as a microbial agent for practical application in soil amelioration, and to systematically investigate the relationship between soil, plants and microorganisms by combining the changes in the soil environment before and after the application of DY1-3 with the results of the growth and physiological indexes of maize plants. We hope to provide a scientific basis and technical support for the evaluation of the practical application of microbial agents, as well as the improvement of soil, growth control of crops, and sustainable development of agriculture.

## 2. Materials and Methods

### 2.1. Test Strains and Experimental Materials

The test strain was derived from the strain *Pseudomonas cedrina* DY1-3, which was isolated and identified from the soil under the moss community of Tian Shan Glacier No. 1 (coordinates: 86°49′ E, 43°05′ N) in the laboratory in the preliminary stage, and its ACC deaminase activity was 4.734 ± 0.037 U/mg as determined by the preliminary enzyme activity [30], and later studies on this strain found that it also has a stronger ability to tolerate salt and drought and accumulate EPS, IAA, iron carriers, and other biomasses than other PGPB, and that it can positively affect the growth of maize plants under both low salt and low drought stress [31]. The experimental maize variety was Sweet Glutinous Jade 788. The medium was trypticase soy broth (TSB): tryptic peptone, 17 g; soy peptone, 3 g; sodium chloride, 5 g; glucose, 2.5 g; dipotassium hydrogen phosphate, 2.5 g; distilled water, 1 L; pH 7.3; and autoclaved by heating at 121 °C for 20 min.

### 2.2. Soil Sample Collection

Shuimogou District (87°47′ E, 43°51′ N), Urumqi City, was selected as the sampling area, and two types of soils, maize arable soil and mildly saline soil, were collected, which were divided into four groups according to the way of subsequent experimental use. The soil was divided into four groups according to the way of subsequent experiments. They were original maize arable soil (OG) and original salinised soil (OY), which were directly used for the sequencing of soil bacterial 16S rRNA V3-V4 region amplicons; maize arable soil (GA) and salinised soil (YA), which were used for the potting experiments and irrigated with DY1-3 bacterial suspension; and maize arable soil (GB) and salinised soil (YB), which were used for potting experiments and irrigated with aseptic water (YB).

**Figure 1:**
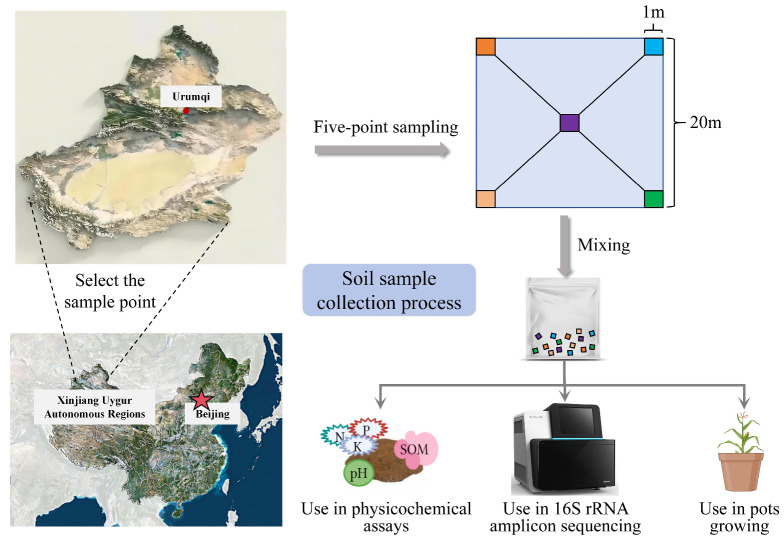
Illustration of the soil sample collection process and how it is utilised. Two types of soils, maize arable soil and mild saline soil, were collected from Shuimogou District, Urumqi, Xinjiang (87°47′ E, 43°51′ N) using a five-point sampling method, and these soils were subsequently used for the determination of the physical and chemical properties of the soils, sequencing of the 16S rRNA amplicons, and potting experiments.

Soil collection with reference to the method of Sun D et al. [32]. The specific sampling process is shown in Figure 1. In the sampling area, a 20 m × 20 m soil sampling area was selected, and the five-point sampling method was used to mark the four corners and the middle of the soil sampling area, and a 1 m × 1 m soil sampling block was divided in the marked area. Wearing disposable gloves, we scraped the surface of the soil collection area to reveal a flat surface and used a small, sterilised shovel to take a sufficient amount of soil samples from each soil collection area and then mixed the samples taken at the five points. The mixed soil samples were grouped according to the utilisation method described in the previous section, and OG and OY were preserved on dry ice and sent to Beijing Ovison Gene Science and Technology Co., Ltd. (Beijing, China) for the determination of soil physico-chemical properties and sequencing of soil bacterial 16S rRNA V3-V4 region amplicons, and soil samples of the GA, GB, YA, and YB groups were brought back to the laboratory for potting and planting. The collection time was October 2023 at the end of the maize harvest.

### 2.3. Determination of Soil Physical and Chemical Properties

Soil pH was determined by potentiometric method; soil total salt (TS) content was determined by mass method; total nitrogen (TN) content was determined by AA3 flow-through assay; available phosphorous (AP) content was determined by molybdenum-antimony colorimetric method; available potassium (AK) content was determined by flame photometry; soil organic matter (SOM) content was determined by potassium dichromate-concentrated sulphuric acid external heating method [33]. This step was performed by Beijing Ovison Gene Science and Technology Co., Ltd. (Beijing, China).

### 2.4. Sequencing of Amplicons of Soil Bacterial 16S rRNA V3-V4 Region

Extraction of genomic DNA from soil samples was carried out using the E.Z.N.A. Soil DNA Kit (Omega Bio-tek, Inc., Norcross, GA, USA), followed by detection of DNA quality and concentration using the Nanodrop 2000 (Thermo Fisher Scientific, Inc., Waltham, MA, USA). Primers 338F (5′-ACTCCTACGGGGAGGCAGCAG-3′) and 806R (5′-GGACTACHVGGGTWTCTAAT-3′) were used to amplify the bacterial 16S rRNA gene PCR reaction system (25 μL): 2 μL of DNA template (total amount of DNA added was 30 ng), 1 μL of forward primer (5 μM), 1 μL of reverse primer (5 μM), 3 μL of BSA (2 ng/μL), 12.5 μL of Taq Plus Master Mix (2×), and 5.5 μL of ddH_2_O. PCR amplification parameters: 95 °C for 5 min; 95 °C for 45 s, 55 °C for 50 s, 72 °C for 45 s, cycling 28 times; 72 °C for 10 min [34]. The amplification products were purified with the Agencourt AMPure XP (Beckman Coulter, Inc., Brea, CA, USA) Nucleic Acid Purification Kit. The amplicon libraries were sequenced using the Illumina NextSeq 2000 platform at Beijing Ovison Gene Science and Technology Co., Ltd. (Beijing, China).

### 2.5. Potting Experiment

The strain and seed treatment work were carried out first. *Pseudomonas cedrina* DY1-3 was inoculated into TSB medium at an inoculum of 2% and incubated at 30 °C, 180 rpm, until plateau stage. A 6500 rpm centrifugation for 10 min was used to obtain the bacterial precipitate, and the bacterial precipitate was washed by adding an appropriate amount of sterile water, and the washing was repeated three times. The bacterial suspension was prepared by adding sterile water to adjust the concentration of bacterial suspension to OD_600_ = 1.0. Maize seed treatment was carried out using the method of Zhao Longfei et al. for seed selection and sterilisation [35]. This was performed by selecting maize seeds with full and undamaged surfaces, rinsing the seeds several times with distilled water to remove surface dust, then rinsing the seeds twice with sterile water, and then soaking the seeds twice in 75% ethanol for one minute each time, then soaking the seeds for five minutes in sodium hypochlorite, and finally rinsing the seeds three to five times with sterile water. Thoroughness of the antiseptic treatment was observed by incubation on beef paste peptone medium coated with sterile water used for the last rinsing. The sterilised maize seeds were soaked in sterile water for 10 h for subsequent seedling establishment.

The next step was the seedling nursery and pot transplanting in hole trays. The treated maize seeds were transferred into seedling trays and grown to the three-leaf stage (light intensity 4000 Lux, light 16 h/d, temperature 30 °C). The soil was mixed with vermiculite in the ratio of 3:1 and sterilised at 121 °C for 20 min. Maize seedlings grown to the three-leaf stage were transplanted. A total of 15 mL of bacterial suspension was applied for the first time after 3 d, and sterile water was applied to the control group. Thereafter, 5 mL of bacterial suspension was applied every 3 d, and an equal amount of sterile water was applied to the control group. Soil moisture content was measured daily and kept within the normal soil moisture content range (18–25%). Three replicates were set up for each set of experiments. The potting culture was completed after 30 d of potting.

The final task of the potting experiment was the determination of the relevant indicators. Relative plant height, relative stem thickness, relative root meristem number, and relative primary root length of potted plants were determined using vernier callipers and other tools. And three different leaf positions were collected for the determination of chlorophyll content, peroxidase (POD), catalase (CAT), superoxide dismutase (SOD), and malondialdehyde (MDA). The above physiological indexes were determined by using the reagent kit method, which was purchased from Suzhou Grace Biotechnology Co. (Suzhou, China). The soil was collected from the inter-root of potted plants, preserved in dry ice, and sent to Beijing Ovison Gene Technology Co., Ltd. (Beijing, China) for the determination of soil physico-chemical properties and sequencing of soil bacterial 16S rRNA V3-V4 amplicons.

### 2.6. Data Analysis

We used GraphPad^®^ Prism v9.0 (GraphPad Software, San Diego, CA, USA) to create a histogram and used a one-way ANOVA and Tukey’s post hoc test to test the significance of the difference (*p* < 0.05) by SPSS v. 23 software (IBM, Armonk, NY, USA); the results were expressed as the mean ± the standard deviation for each sample point. The analysis of soil bacterial community diversity and the correlation analysis between soil bacterial species and environmental factors were carried out using the Ovison computer platform, and the sequencing platform analysis tools were used for the drawing of Venn diagrams, PCA analysis diagrams, and RDA analysis diagrams.

## 3. Results

### 3.1. Physico-Chemical Properties of Virgin Soil and Sequencing Analysis of 16S rRNA V3-V4 Region Amplicon of Soil Bacteria

#### 3.1.1. Physico-Chemical Properties of the Original Soil

**Figure 2:**
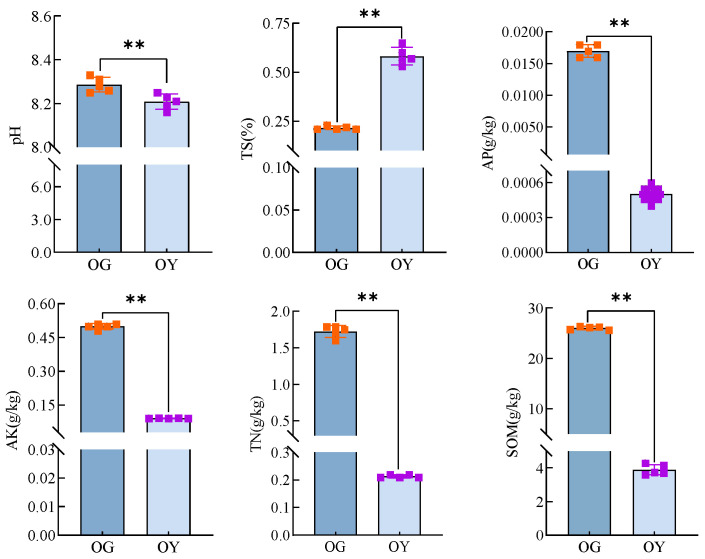
Results of raw soil physico-chemical property measurements. ** indicates highly significant difference (*p* < 0.01).

The results of different soil physicochemical property measurements are shown in Figure 2. Both soils are alkaline soils [33], with mean pH values ranging from 8.21 to 8.29, and the pH level of arable soils is significantly higher than that of saline soils (*p* < 0.01); the mean values of the TS content of the two types showed a highly significant difference (*p* < 0.01), with the TS content of the OY group being (0.58 ± 0.04) g/kg, which is a mildly saline soil; from the nutrient results, it can be seen that AP, AK, TN, and SOM had the same trend, all of which showed that their contents were significantly higher in arable soils than in saline soils (*p* < 0.01).

#### 3.1.2. Raw Soil Data Pre-Processing Statistics, Quality Control, and OUT Distribution Analysis 

**Figure 3:**
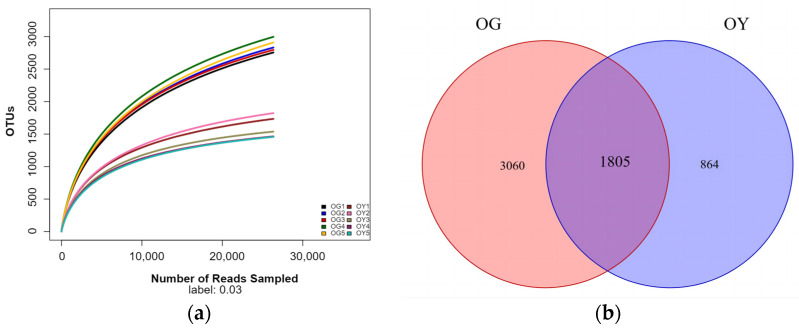
Dilution curves and distribution of OTUs for different types of soil samples; (**a**) is a graph of the dilution curves; (**b**) is a Venn diagram of the OTUs distribution.

Based on the amplicon sequencing results (Table 1), the Clean Reads of the five arable soil samples ranged from 53,284 to 72,497, with valid sequences accounting for 96.35–97.74% of the total sequences, and the OUT numbers ranged from 2755–2996. The Clean Reads of the five saline soil samples ranged from 27,431 to 56,307, with valid sequences accounting for 99.02–99.39% of the total sequences, and the OUT numbers ranged from 1456 to 1822. From the dilution curves of the soil samples (Figure 3a), it can be seen that the dilution curves of the 10 soil samples gradually flattened out, indicating that the OTUs in this environment do not increase significantly with the increase in the sample size, which suggests that the amount of sequencing data is reasonable.

Venn diagrams were used to analyse the composition of shared and endemic bacterial OTUs in both soil types to visualise similarities and overlaps. It can be seen that a total of 5729 OTUs were found in the two types of soils, of which 4865 OTUs, or 84.92% of the total, were detected in OG and 2669 OTUs, or 46.59% of the total, were detected in OY; the number of OTUs common to the two types of soils was 1805, or 31.51% of the total; and there were OTUs unique to the two types of soils, with 3060 OTUs, or 53.41% of the total, unique to OG, and there were OTUs unique to the two types of soils. OTUs unique to OG were 3060, accounting for 53.41% of the total, and OTUs unique to OY were 864, accounting for 15.8% of the total. It can be seen that the total OTUs and unique OTUs in the arable soil were significantly higher than those in the saline soil.

#### 3.1.3. Analysis of Alpha Diversity Indices of Pristine Soil Bacterial Communities

The diversity of bacterial communities in the two soils was analysed, and the results of the OTUs, Shannon index, Simpson index, and Chao1 index are shown in Table 2. The sequencing coverage of the two soil samples was 0.9652 ± 0.0018 and 0.9861 ± 0.0027, respectively, which were close to 1, indicating that the sequencing results could represent the real situation of microorganisms in the samples. The pattern of change in the OTUs number, Shannon index, Simpson index, and Chao1 index was consistent in both soil samples, which showed that they were significantly higher in OG than in OY (*p* < 0.05). Overall, the diversity and richness of soil bacterial community in arable soil was higher than that in saline soil.

#### 3.1.4. Analysis of the Composition of the Original Soil Bacterial Community

**Figure 4:**
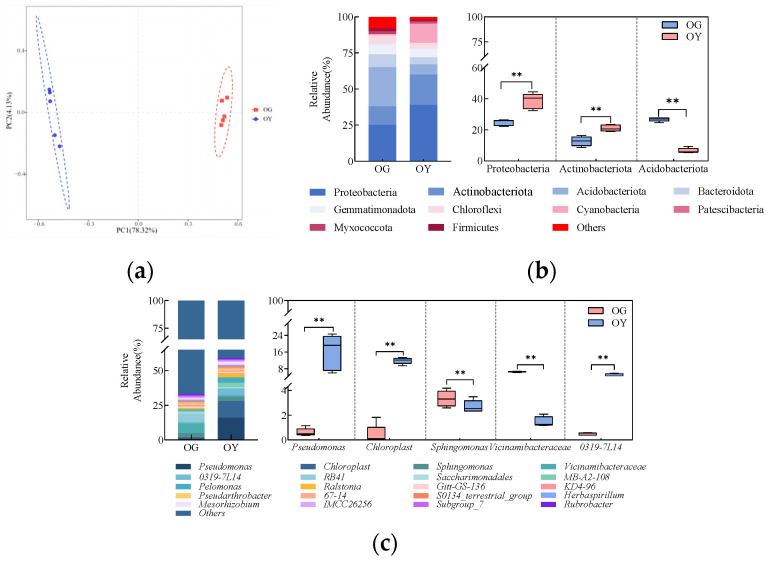
Analysis of the composition of the original soil bacterial community; (**a**) graph of PCA analysis; (**b**) significance analysis of the horizontal composition of the two soil bacterial phyla and the top three dominant phyla, ** indicates highly significant differences (*p* < 0.01); (**c**) significance analysis of the genus-level composition of the two soil bacteria and the top five dominant genera, ** indicates highly significant differences (*p* < 0.01).

The results of Principal Component Analysis (PCA) of bacterial communities in both types of soils are shown in Figure 4a. The differences and distances between the samples were reflected by analysing the OUT (97% similarity) composition of different samples, and the more similar the composition of the samples, the closer they are to each other. PC1 and PC2 explained 78.32% and 4.31% of the differences between the samples, respectively, for a total of 82.63%. From the figure, it can be seen that five samples of the same type of soil can be well duplicated together, and the distribution areas of OG and OY are clearly distinguished, indicating that there are differences in the structure of the bacterial community in the two types of soils.

The top 10 bacterial phyla in terms of relative abundance in both soil bacterial communities are shown in Figure 4b, with the major phyla including Proteobacteria, Actinobacteriota, and Acidobacteria. Among them, Proteobacteria was the most dominant phylum, with relative abundance ranging from 24.74% to 38.65%, followed by Actinobacteriota, with relative abundance ranging from 12.66% to 21.05%, and Acidobacteria was mainly enriched in OG, with an average relative abundance of 26.72% in OG, an average relative abundance of 26.72%. The top three dominant phyla were further analysed for significance of differences. The results showed that the relative abundance of Proteobacteria and Actinobacteriota was significantly (*p* < 0.01) higher in OY, and the relative abundance of Acidobacteria was significantly (*p* < 0.01) higher in OG.

The top 20 bacterial genera in terms of relative abundance at the genus level in both soil bacterial communities are shown in Figure 4c. The dominant genera included Pseudomonas, Chloroplast, Sphingomonas, Vicinamibacteraceae, and 0319-7L14. Vicinamibacteraceae was the dominant taxon in the OG, with an average relative abundance of 6.62% in the OG, and Pseudomonas was the dominant taxon in the OY, with an average relative abundance of 16.17% in the OY. The top five dominant genera were further analysed for significance of differences. The results showed that the relative abundance of three genera (Pseudomonas, Chloroplast, and 0319-7L14) were significantly higher in OY than in OG (*p* < 0.01); two genera (Sphingomonas and Vicinamibacteraceae) had significantly higher relative abundance in OG than in OY (*p* < 0.01). These results indicated that there were significant differences in the bacterial community composition between the two soil samples.

#### 3.1.5. Analysis of Primary Soil Bacterial Community Structure and Environmental Factors

**Figure 5:**
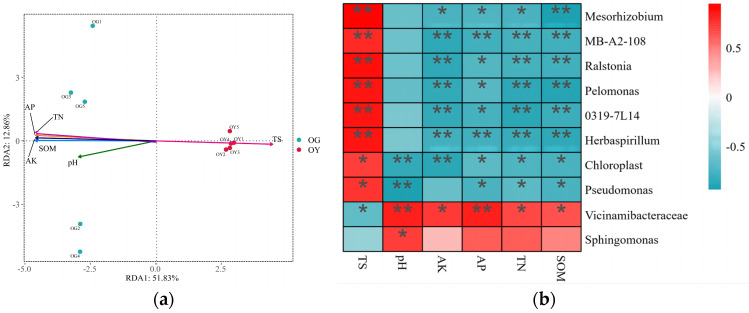
RDA analysis and Spearman correlation analysis of the structural composition of soil bacterial communities with soil physico-chemical factors for the two types of soil bacteria; (**a**) RDA analysis; (**b**) Spearman’s correlation analysis, * denotes a significant difference (*p* < 0.05); ** denotes a highly significant difference (*p* < 0.01).

The results of RDA analysis between the structural composition of the two types of soil bacterial communities and soil physicochemical factors based on the results of soil physicochemical property measurements are shown in Figure 5a (permutation test: *p* = 0.006). The first and second axes demonstrated 51.83% and 12.86% of the explained amount, respectively, indicating that the environmental factors selected for this study were representative. The results showed a highly significant positive correlation between TS and soil microorganisms (R^2^ = 0.9655, *p* = 0.006); AP (R^2^ = 0.9950, *p* = 0.002), AK (R^2^ = 0.9980, *p* = 0.0005), TN (R^2^ = 0.9968, *p* = 0.001), SOM (R^2^ = 0.9929, *p* = 0.007), and pH (R^2^ = 0.6589, *p* = 0.019) were significantly or highly significantly negatively correlated with soil microorganisms. The top 10 dominant bacterial genera at the genus level were selected for Spearman correlation analysis with soil physico-chemical factors to explain the correlation between dominant bacterial genera and soil physico-chemical factors (Figure 5b). The results showed a significant relationship between dominant bacterial genera and soil physico-chemical factors. Specifically, six bacterial genera, *Mesorhizobium*, *MB-A2-108*, *Ralstonia*, *Pelomonas*, *0319-7L14* and *Herbaspirillum*, showed highly significant positive correlations (*p* < 0.01) with soil total salt (TS), and significant or highly significant negative correlations (*p* < 0.01 or *p* < 0.05); *Chloroplast* showed a significant positive correlation (*p* < 0.05) with TS and a significant or highly significant negative correlation (*p* < 0.05 or *p* < 0.01) with soil pH, AK, AP, TN, and SOM; *Pseudomonas* showed a significant positive correlation (*p* < 0.05) with TS and a significant or highly significant negative correlation (*p* < 0.05) with soil pH, AP, TN, and SOM (*p* < 0.05 or *p* < 0.01); while *Vicinamibacteraceae* showed significant negative correlation with TS (*p* < 0.05) and significant or highly significant positive correlation with soil pH, AK, AP, TN, and SOM (*p* < 0.05 or *p* < 0.01); and *Sphingomonas* showed significant positive (*p* < 0.05 or *p* < 0.01) correlation with pH correlation (*p* < 0.05).

### 3.2. Investigating the Effects of the Application of DY1-3 Bacterial Suspension in Arable Soil on the Growth of Maize Seedlings and the Structure of Soil Bacterial Communities

#### 3.2.1. Analysis of the Results of the Determination of Growth and Physiological Indexes of Potted Plants in Arable Soil

**Figure 6:**
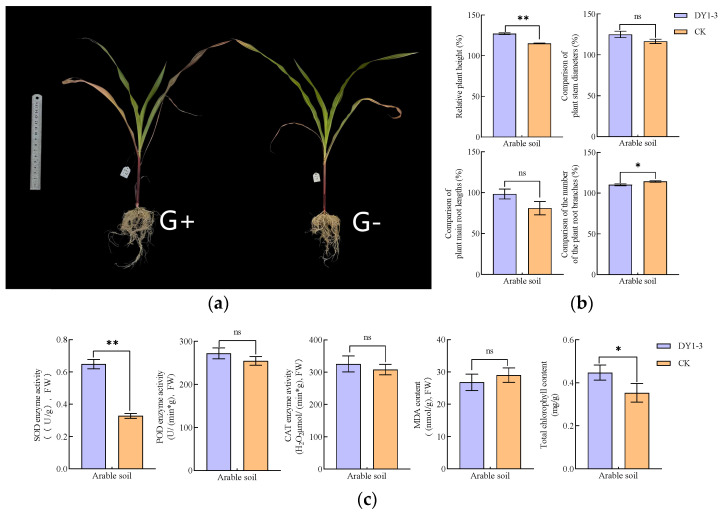
Effect of DY1-3 bacterial suspension on morphological indexes of maize plants cultivated in arable soil for thirty days. (**a**) Photographs of maize seedlings cultivated in arable soil for thirty days with different treatments, G+ denotes the group treated with DY1-3 bacterial suspension during the growth period of the maize seedlings; G- denotes the group treated with sterile water during the growth period of the maize seedlings; (**b**) results of morphological indexes of maize seedlings under different treatments; (**c**) the results of physiological indexes of maize seedlings under different treatments, DY1-3 denotes the treatment group with bacterial suspension; CK denotes the control group with aseptic water, ns denotes non-significant; * denotes significant difference (*p* < 0.05); ** denotes highly significant difference (*p* < 0.01).

Using arable soil for potting experiments, the results of the effect of DY1-3 bacterial suspension application on the relevant morphological and physiological indexes of maize seedlings are shown in Figure 6. In terms of morphological indexes, the application of DY1-3 bacterial suspension highly significantly (*p* < 0.01) increased the plant height of maize seedlings from 115.12 ± 0.56% to 127.25 ± 1.11% and significantly (*p* < 0.05) reduced the number of root branches from 119.35 ± 3.82% to 117.43 ± 2.29%, as compared with maize seedlings cultured with the application of aseptic water (2.29%). In terms of physiological indexes, the application of DY1-3 bacterial suspension highly significantly (*p* < 0.01) increased the SOD content of maize seedlings from (0.33 ± 0.02) U/g to (0.65 ± 0.03) U/g and the total chlorophyll content of maize seedlings from (0.35 ± 0.04) mg/g to (0.45 ± 2.29) U/g, as compared with that of the maize seedlings cultured with the application of aseptic water. g to (0.45 ± 0.03) mg/g.

#### 3.2.2. Analysis of the Results of the Determination of Physico-Chemical Properties of Soil in Pots of Arable Soil

**Figure 7:**
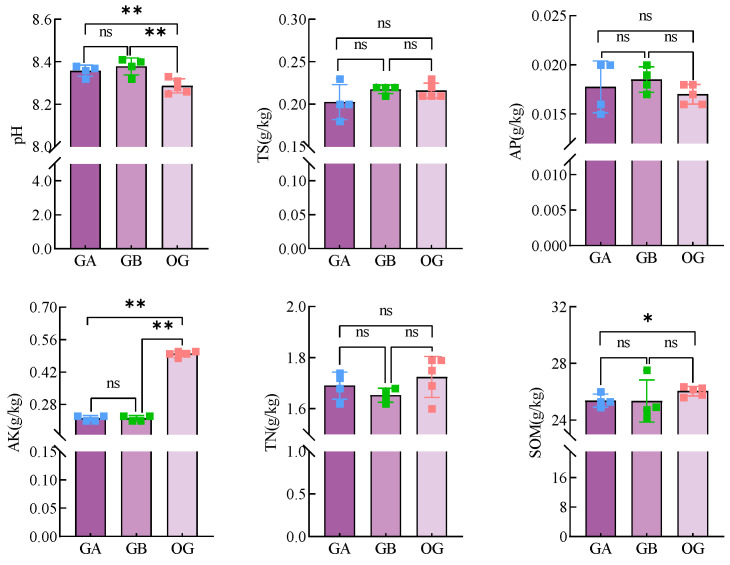
Results of arable soil potting treatments and original soil physico-chemical property measurements; ns indicates no significance; * indicates significant difference (*p* < 0.05); ** indicates highly significant difference (*p* < 0.01).

The results of physicochemical property measurements of arable soil after potting treatment are shown in Figure 7. There was a highly significant (*p* < 0.01) increase in the mean pH of GA and GB from 8.29 to 8.36 and 8.38, and a highly significant (*p* < 0.01) decrease in AK from (0.50 ± 0.01) g/kg to (0.22 ± 0.01) g/kg compared to OG. There was a highly significant (*p* < 0.05) decrease in SOM of GA compared to OG from (26.04 ± 0.32) g/kg to (25.38 ± 0.46) g/kg.

#### 3.2.3. Statistics on Pre-Processing of Arable Soil Data After Potting, Quality Control and Analysis of OUT Distribution

**Figure 8:**
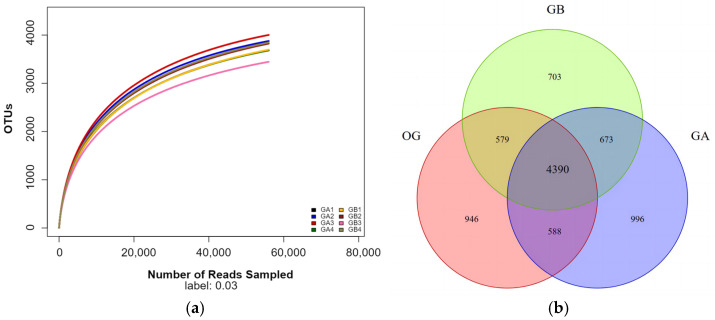
Dilution plot and Venn diagram of OTUs distribution of ploughed soil samples after potting; (**a**) dilution plot; (**b**) Venn diagram of OTUs distribution.

Based on the sequencing results of bacterial 16S rRNA V3-V4 region amplicons (Table 3), the Clean Reads of GA and GB groups ranged from 63,783 to 77,721 and 71,954 to 77,223, respectively, in the potting-treated arable soils, and the distributions of valid sequences to the total sequences ranged from 93.75% to 96.21% and 96.21% to 97.08%, respectively. The ranges of OUT numbers were 3867–4274 and 3662–4022, respectively. As can be seen from the soil sample dilution curves (Figure 8a), the dilution curves of the eight arable land soil samples treated with potting gradually flattened out in both the GA and GB groups, which indicates that the OTUs in this environment do not increase significantly with the increase in the number of samples, suggesting that the amount of sequencing data is reasonable.

Venn diagrams were used to analyse the composition of common and endemic bacterial OTUs in the original soil and the potting soils of the two different treatment groups to provide a visual representation of the similarities and overlaps. As can be seen from Figure 8b, a total of 8875 OTUs were found in the three soils of OG, GA, and GB, of which 6503 OTUs, or 73.27% of the total number, were detected in OG, 6611 OTUs, or 74.49% of the total number, in GA, and 6345 OTUs, or 71.49% of the total number, in GB. The composition of the bacterial OTUs common to the three soil samples of GA, GB, and OG had a total of 4390 OTUs in the three soil samples, accounting for 49.46% of the total. All three groups of soil samples had unique OTUs, including 996 OTUs unique to GA, accounting for 11.22% of the total; 703 OTUs unique to GB, accounting for 7.92% of the total; and 946 OTUs unique to OG, accounting for 10.66% of the total. It can be seen that pot planting changed the flora structure of the original arable soil, which led to changes in the grouping of OTUs. Meanwhile, compared with GB, unique OTUs also appeared in GA, indicating that the application of DY1-3 bacterial suspension could also make changes in the structure of the soil flora.

#### 3.2.4. Analysis of Alpha Diversity Indices of Soil Bacterial Communities in Arable Land After Potting Plants

The diversity of bacterial communities in the original arable soil and the pot-planted arable soil of two different treatment groups was analysed, and the results obtained for the number of OTUs, Shannon index, Simpson index, and Chao1 index are shown in Table 4. The sequencing coverage of the soil samples of the three groups, GA, GB, and OG, were 0.9783 ± 0.0016, 0.9777 ± 0.0011, and 0.9789 ± 0.0009, which tended to be close to 1, indicating that the sequencing results could represent the real situation of microorganisms in the samples. Compared with OG, Shannon and Simpson indices increased significantly (*p* < 0.05) in GA and GB, as shown by the increase in the Shannon index from 9.9436 ± 0.1012 to 10.1499 ± 0.1251 and 10.0085 ± 0.1465, and the Simpson index from 0.9970 ± 0.0002 to 0.9977 ± 0.0002 and 0.9789 ± 0.0009, respectively, indicating that sequencing results could represent the real situation of microorganisms in the samples. to 0.9977 ± 0.0002 and 0.9973 ± 0.0003, and Simpson index increased significantly (*p* < 0.05) from 0.9973 ± 0.0003 to 0.9977 ± 0.0002 in GA compared to GB.

#### 3.2.5. Analysis of Bacterial Community Composition in Arable Soil After Potting

**Figure 9:**
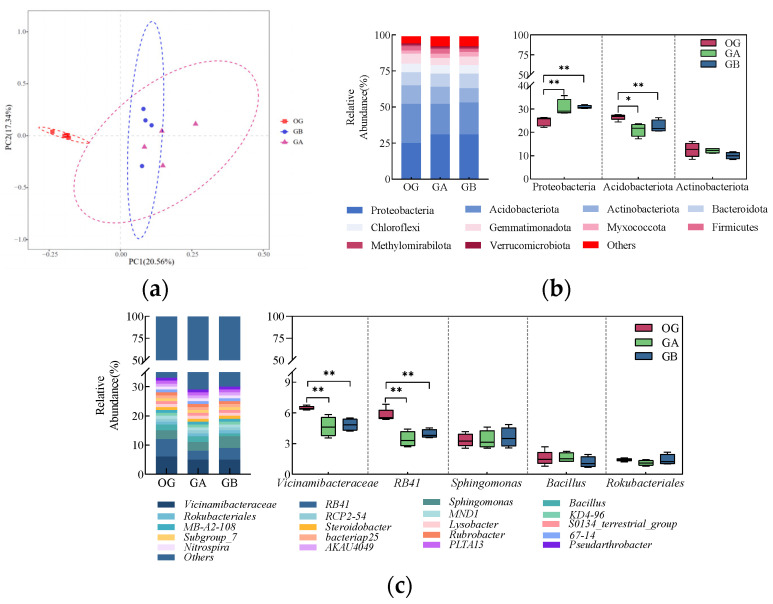
Analysis of the composition of the arable soil bacterial community after potting; (**a**) graph of PCA analysis; (**b**) bacterial phylum level composition and differential analysis of the top three dominant phyla in original arable soil and post-potting arable soil; (**c**) bacterial genus level composition and differential analysis of the top five dominant phyla of soil bacteria in the original arable soil and in the arable soil after potted plants, * indicates significant differences (*p* < 0.05), ** indicates highly significant differences (*p* < 0.01).

The results of the principal component analysis of the bacterial community in the original arable soil and the potted arable soil of two different treatment groups are shown in Figure 9a. The differences and distances between samples were reflected by analysing the OTU (97% similarity) compositions of different samples, and the more similar the sample compositions were, the closer the distances between them were. PC1 and PC2 explained 20.56% and 17.34% of the differences between the samples, respectively, which totalled 37.90%. From the figure, it can be seen that the samples from the three treatment groups of GA, GB, and OG can be better duplicated together, and the distribution areas of OG and potted treatments of GA and GB are clearly differentiated, which indicates that there are differences in the structure of the bacterial community in the original arable soil and the potted arable soil of the two different treatment groups.

The top 10 bacterial phyla in terms of relative abundance in the bacterial communities in the OG, GA, and GB, shown in Figure 9b. The major phyla include Proteobacteria, Acidobacteria, and Actinobacteriota. Among them, Proteobacteria was the most dominant phylum with a relative abundance range of 24.70–30.84%, followed by Acidobacteria with a relative abundance range of 21.15–26.53% and Actinobacteriota with a relative abundance range of 10.01–12.58%. The top three dominant phyla were further analysed for significance of differences. The results showed that the relative fractions of Proteobacteria in GA and GB had a highly significant increase (*p* < 0.01) compared to OG; the relative abundance of Acidobacteria in GA was significantly lower (*p* < 0.05), and the relative abundance of Acidobacteria in GB was highly significant (*p* < 0.01).

The top 20 bacterial genera in terms of relative abundance at the genus level in the bacterial communities in the OG, GA, and GB, shown in Figure 9c. The dominant genera included *Vicinamibacteraceae*, *RB41*, *Sphingomonas*, *Bacillus*, and *Rokubacteriales*. The dominant bacterial genus in the arable soils of the three different treatment groups was *Vicinamibacteraceae*, with an average relative abundance of 6.48%, 4.66%, and 4.85% in OG, GA, and GB, respectively. The top five dominant genera were further analysed for significance of differences. Both *Vicinamibacteraceae* and *RB41* were highly significantly lower (*p* < 0.01) in GA and GB compared to OG.

#### 3.2.6. RDA Analysis Between Soil Bacterial Community Structure Composition and Soil Physicochemical Factors in Arable Soil

**Figure 10:**
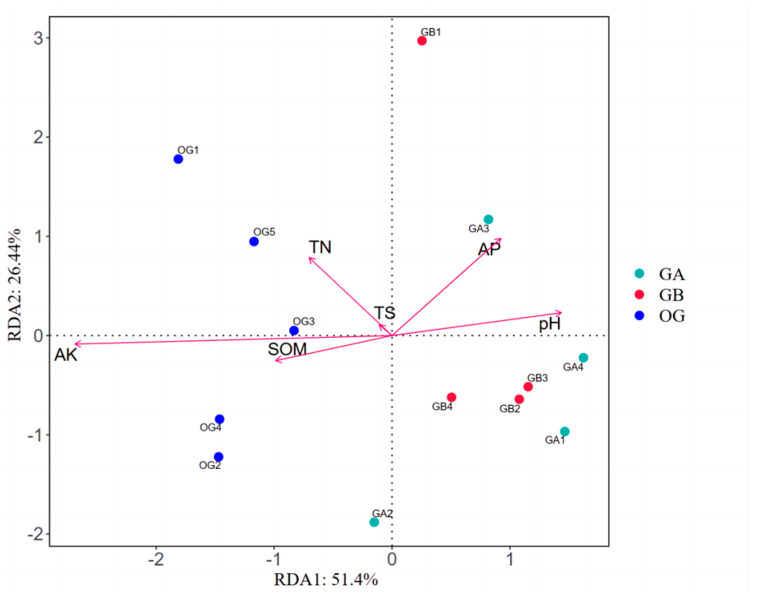
RDA analysis of soil bacterial community structural composition and soil physico-chemical factors in the original arable soil and in the arable soil after potting.

Based on the results of soil physicochemical property measurements, the results of RDA analysis between soil bacterial community structural composition and soil physicochemical factors of arable soil after potting treatment and original arable soil are shown in Figure 10 (permutation test: *p* = 0.014). The first and second axes demonstrated 51.40% and 26.44% of the explained amount, respectively, indicating that the environmental factors selected for this study were representative. The results indicated that AK was highly significant and negatively correlated with soil microorganisms (R^2^ = 0.8043, *p* = 0.001) and was a key factor influencing the structural composition of bacterial communities in the original arable soil and in the arable soil after potting treatment.

### 3.3. Investigating the Effect of the Application of DY1-3 Bacterial Suspension in Saline Soil on the Growth of Maize Seedlings and the Structure of Soil Bacterial Community

#### 3.3.1. Measurement Results of Growth and Physiological Indexes of Potted Plants in Saline Soil

**Figure 11:**
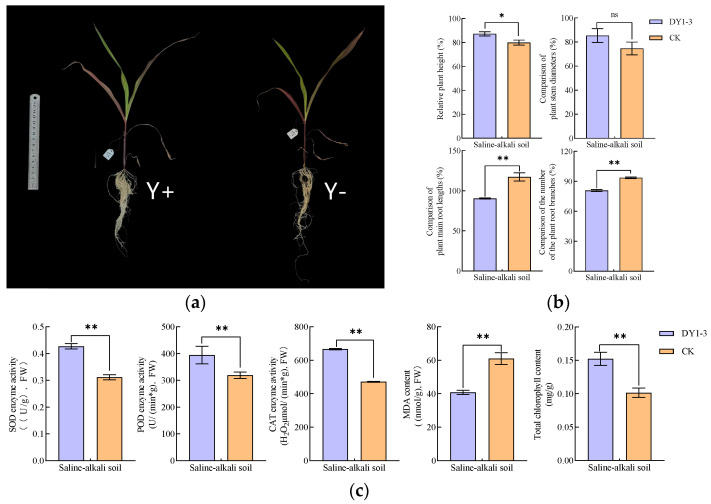
Effect of DY1-3 bacterial suspension on morphological indexes of maize seedlings cultivated in saline soil for thirty days. (**a**) Photographs of maize seedlings cultivated in saline soil with different treatments for thirty days; Y+ denotes the group treated with DY1-3 bacterial suspension during the growth period of the maize seedlings; Y- denotes the group treated with sterile water during the growth period of the maize seedlings. (**b**) Results of the determination of the morphological indexes of the maize seedlings with different treatments. (**c**) Results of the determination of physiological indexes of the maize seedlings with different treatments; DY1-3 denotes the treatment group with bacterial suspension; CK denotes the control group with aseptic water; ns denotes non-significant; * denotes significant difference (*p* < 0.05); ** denotes highly significant difference (*p* < 0.01).

Using saline soil for potting experiments, the results of the effects of DY1-3 bacterial suspension application on the relevant morphological and physiological indexes of maize seedlings are shown in Figure 11.

In terms of morphological indexes, the application of DY1-3 bacterial suspension significantly (*p* < 0.05) increased the plant height of maize seedlings compared with that of maize seedlings cultured with sterile water, with the relative plant height increasing from 82.93 ± 3.01% to 87.20 ± 2.16%; and it very significantly (*p* < 0.01) reduced the primary root length and the number of root branches of maize seedlings, which was reflected by the reduction of the relative primary root length from 117.16 ± 5.11% to 90.45 ± 2.16%, and the relative primary root length from 117.16 ± 5.11% to 90.45 ± 2.16%, respectively; and 5.11% to 90.45 ± 0.73%, and relative root branching number from 89.90 ± 3.42% to 84.56 ± 2.94%. In terms of physiological indexes, the application of DY1-3 bacterial suspension highly significantly (*p* < 0.01) increased the SOD, POD, CAT and total chlorophyll contents of maize seedlings compared to those cultured with sterile water, as shown by the increase in the SOD content from (0.31 ± 0.01) U/g to (0.43 ± 0.01) U/g, POD content from (319.07 ± 11.96) U/(min·g) to (394.57 ± 33.16) U/(min·g), CAT content from (471.55 ± 2.85) μmol/(min·g) to (666.82 ± 4.25) μmol/(min·g), total chlorophyll content from (0.10 ± 0.01) mg/g to (0.15 ± 0.01) mg/g; and the application of DY1-3 bacterial suspension highly significantly (*p* < 0.01) reduced the MDA content of maize seedlings from (60.02 ± 3.55) nmol/g to (40.78 ± 1.29) nmol/g.

#### 3.3.2. Results of Physicochemical Property Determination of Soil in Potting Saline Soil

**Figure 12:**
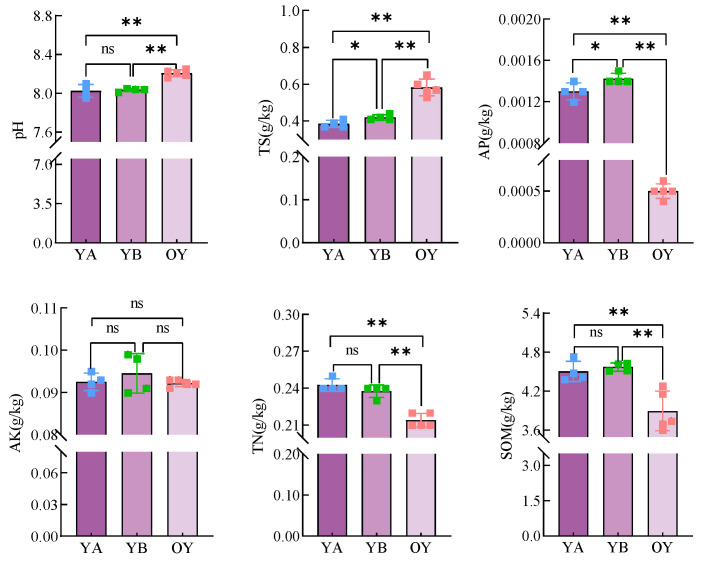
Results of potting treatments and original soil physico-chemical property measurements on saline soils; ns indicates no significance; * indicates significant difference (*p* < 0.05); ** indicates highly significant difference (*p* < 0.01).

The results of physicochemical property determination of saline soil after potting treatment are shown in Figure 12. Compared with OY, there were significant changes in all physicochemical indicators in saline soil after potting treatment. Specifically, the average pH of YA and YB was highly significant (*p* < 0.01) lower than that of OY, from 8.21 to 8.02 and 8.03; TS was highly significant (*p* < 0.01) lower than that of OY, from (0.58 ± 0.05) g/kg to (0.38 ± 0.02) g/kg and (0.42 ± 0.02) g/kg; and AP was highly significant (*p* < 0.02) higher than that of OY. significantly (*p* < 0.01) higher than OY from (0.49 ± 0.08) g/kg to (1.30 ± 0.09) g/kg and (1.42 ± 0.03) g/kg; TN was highly significant (*p* < 0.01) higher than OY from (0.21 ± 0.01) g/kg to (0.24 ± 0.01) g/kg and (0.23 ± 0.01) g/kg; SOM was highly significantly (*p* < 0.01) increased from (3.89 ± 0.30) g/kg to (4.50 ± 0.15) g/kg and (4.57 ± 0.07) g/kg compared to OY. and the application of DY1-3 bacterial suspension also significantly (*p* < 0.05) reduced the TS and AP contents in the potting soil compared to GB.

#### 3.3.3. Pre-Processing Statistics, Quality Control and OUT Distribution Analysis of Saline Soil Data After Potting Treatment

**Figure 13:**
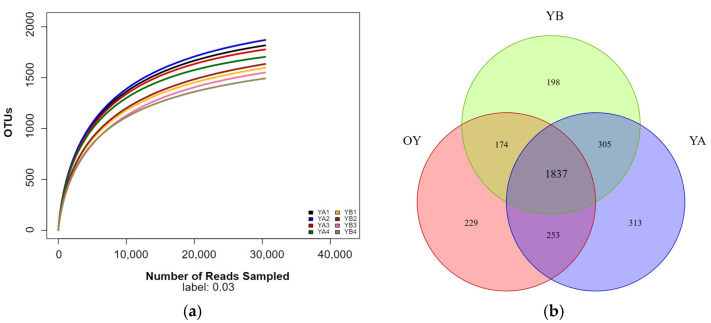
Dilution plot and Venn diagram of OTUs distribution of saline soil samples after potting. (**a**) is the dilution plot. (**b**) is the Venn diagram of OTUs distribution.

Based on the amplicon sequencing results (Table 5), in the saline soil after potting treatment, the Clean Reads of YA and YB ranged from 31,988 to 44,645 and 32,136 to 70,647, respectively, with valid sequences accounting for 97.96–98.10% and 94.78–95.56% of the total sequences, and the ranges of the OUT numbers were 1772–1927 and 1543–1709. From the soil sample dilution curves (Figure 13a), it can be seen that in the eight saline soil samples treated with potting, the dilution curves gradually flattened in both the YA and YB groups, indicating that the OTUs in this environment do not increase significantly with the increase in the number of samples, which suggests that the amount of sequencing data is reasonable.

Venn diagrams were used to analyse the composition of common and endemic bacterial OTUs in the original soil and in the potting soil of the two different treatment groups to provide a visual representation of the similarities and overlaps. As can be seen from Figure 13b, a total of 3309 OTUs were found in OY, YA, and YB, of which 2493 OTUs, or 75.34% of the total number, were detected in OY, 2708 OTUs, or 81.84% of the total number in YA, and 2514 OTUs, or 75.97% of the total number in YB. The three types of soil samples, YA, YB, and OY, had a total of 1837 OTUs or 55.51% of the total. All three groups of soil samples had unique OTUs, including 313 OTUs unique to YA, accounting for 9.46% of the total; 198 OTUs unique to YB, accounting for 5.98% of the total; and 229 OTUs unique to OY, accounting for 6.92% of the total. It can be seen that pot planting changed the flora structure of the original saline soil, which led to changes in the grouping of OTUs. At the same time, the total OTUs in YA were significantly higher than those in OY and YB, indicating that the application of DY1-3 bacterial suspension could also make changes in the structure of the soil flora.

#### 3.3.4. Analysis of Alpha Diversity Indices of Bacterial Communities in Saline Soils After Potting

The bacterial community diversity in OY, YA, and YB was analysed, and the results obtained for the number of OTUs, Shannon index, Simpson index, and Chao1 index are shown in Table 6. The sequencing coverages of soil samples in the YA, YB, and OY groups were 0.9851 ± 0.0007, 0.9858 ± 0.0010, and 0.9870 ± 0.0020, respectively, which converged to 1, indicating that the sequencing results can represent the real situation of microorganisms in the samples. Compared with OY and YB, the OTUs, Shannon index, and Chao1 index of YA increased significantly (*p* < 0.05), as shown by the increase in OTUs from 1578 ± 156 and 1613 ± 70 to 1858 ± 68; the Shannon index from 7.5832 ± 0.5175 and 7.9511 ± 0.2400 to 8.7032 ± 0.1808; and the Chao1 index from 7.5832 ± 0.5175 and 7.9511 ± 0.2400 to 8.7032 ± 0.1808. The Chao1 index increased from 1821.878 ± 190.620 and 1894.056 ± 103.166 to 2120.417 ± 66.787. and there was a significant (*p* < 0.05) increase in both Simpson and Chao1 indices in YA and YB compared to OY, as evidenced by an increase in the Simpson index from 0.9613 ± 0.0200 to 0.9895 ± 0.0039 and 0.9812 ± 0.0052. The Chao1 index increased from 1821.878 ± 190.620 to 2120.417 ± 66.787 and 1894.056 ± 103.166.

#### 3.3.5. Analysis of Bacterial Community Composition of Saline Soil After Potting

**Figure 14:**
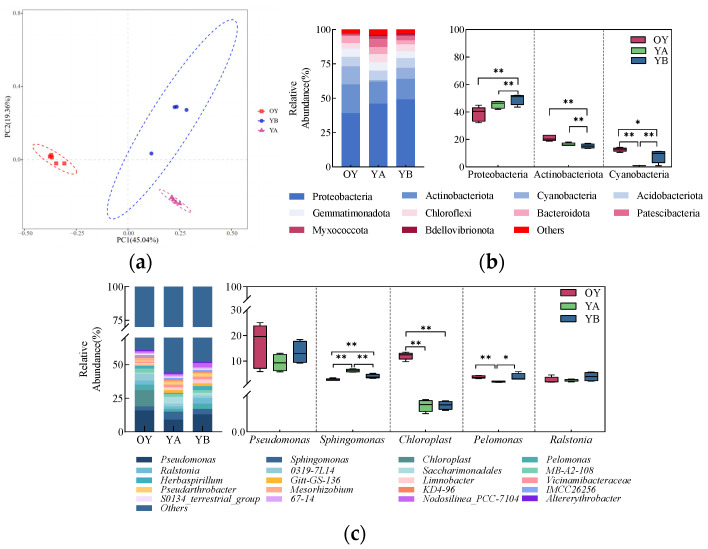
Analysis of the composition of the saline soil bacterial community after potting; (**a**) graph of PCA analysis; (**b**) bacterial phylum level composition and differential analysis of the top three dominant phyla in original saline soil and post-potting saline soil; (**c**) bacterial genus level composition and differential analysis of the top five dominant phyla of soil bacteria in the original saline soil and in the saline soil after potted plants, * indicates significant differences (*p* < 0.05), ** indicates highly significant differences (*p* < 0.01).

The results of principal component analysis of bacterial communities in OY, YA, and YB are shown in Figure 14a. The differences and distances between samples were reflected by analysing the OTU (97% similarity) composition of different samples, and the more similar the sample compositions were, the closer they were to each other. PC1 and PC2 explained 45.04% and 19.36% of the sample differences, respectively, totalling 64.40%. From the figure, it can be seen that the samples from the three treatment groups of YA, YB, and OY can be better duplicated together, and the distribution areas of the three groups are clearly differentiated, indicating that there are differences in the structure of the bacterial communities in the three soils of the original OY, YB, and YA.

The top 10 bacterial phyla in relative abundance in OY, YA, and YB are shown in Figure 14b. The major phyla include Proteobacteria, Actinobacteria, and Cyanobacteria. Proteobacteria was the most dominant phylum, with relative abundance ranging from 38.63 to 49.46%, followed by Actinobacteria, with relative abundance ranging from 15.21 to 20.94%, and Actinobacteria were mainly enriched in OY and YB, with relative abundance ranging from 12.72% and 8.04%, respectively. The top three dominant phyla were further analysed for significance of differences. The results showed that compared with OY, the relative abundance of Proteobacteria in YA and YB had a highly significant increase (*p* < 0.01); the relative fraction of Actinobacteria, an Actinobacteria phylum, had a highly significant decrease (*p* < 0.01); and the relative fraction of Cyanobacteria had a significant decrease (*p* < 0.05). And there was a highly significant decrease (*p* < 0.01) in the relative scores of Cyanobacteria in YA compared to OY.

The top 20 bacterial genera in terms of relative abundance at the genus level in OY, YA, and YB are shown in Figure 14c. The dominant genera included *Pseudomonas*, *Sphingomonas*, *Chloroplast*, *Pelomonas*, and *Ralstonia*. The dominant genus in saline soils of the three different treatment groups was *Pseudomonas*, with average relative abundances in OY, YA, and YB of 16.26%, 9.33%, and 13.37%. The top five dominant genera were further analysed for significance of differences. The results showed that the relative abundance of *Sphingomonas* was highly significantly higher (*p* < 0.01) and that of *Chloroplast* was highly significantly lower (*p* < 0.01) in YA and YB compared to OY. Compared to YB, the relative abundance of *Sphingomonas* in YA was highly significantly increased (*p* < 0.01); the relative abundance of *Pelomonas* was significantly decreased (*p* < 0.05).

#### 3.3.6. RDA Analysis of the Structural Composition of Saline–Alkaline Soil Bacterial Community and Soil Physicochemical Factors

**Figure 15:**
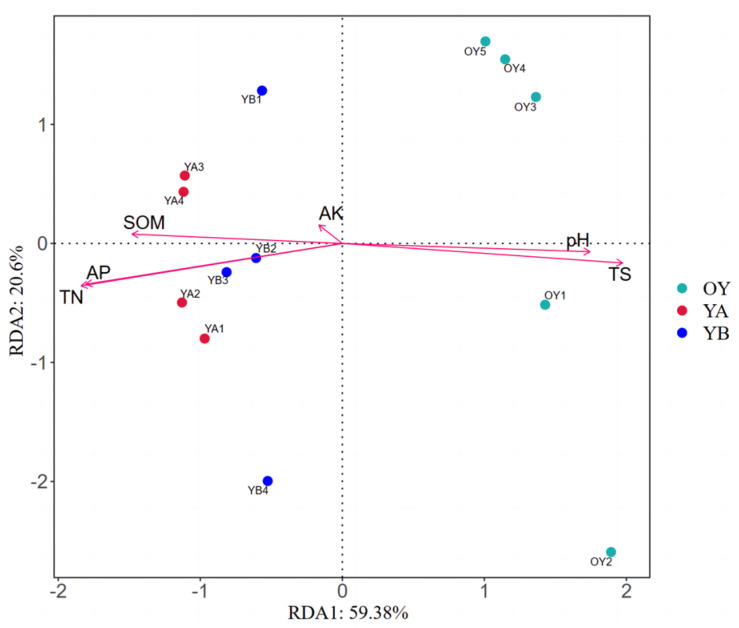
RDA analysis of bacterial community structure composition and soil physico-chemical factors in original saline soil and post-potting saline soil.

Based on the results of soil physicochemical property measurements, RDA analyses between the structural composition of bacterial community and soil physicochemical factors in saline and original saline soil after potting treatments were performed, and the results are shown in Figure 15 (permutation test: *p* = 0.001). The first and second axes demonstrated 59.38% and 20.6% of the explained amount, respectively, indicating the representativeness of the environmental factors selected for this study. The results showed that pH (R^2^ = 0.8620, *p* = 0.001) and TS (R^2^ = 0.9781, *p* = 0.001) were highly significant positively correlated with soil microorganisms, while AP (R^2^ = 0.9076, *p* = 0.001), TN (R^2^ = 0.9250, *p* = 0.001), and SOM (R^2^ = 0.7323, *p* = 0.001) showed a highly significant negative correlation with soil microorganisms.

### 3.4. Investigate the Effects of DY1-3 Strain Application and Planting of Maize Seedlings on Soil Improvement in Saline and Alkaline Soils

Comparative analysis of soil physico-chemical properties, OUT distribution analysis, and PCA analysis were used to explore the effects of DY1-3 strain application and planting of maize seedlings on the nutrient content and microbial community composition of saline–alkaline soils in order to analyse the improvement of saline–alkaline soils.

**Figure 16:**
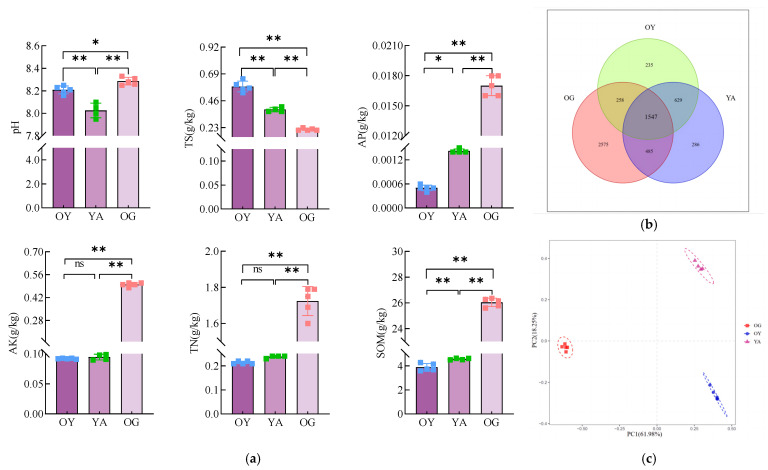
Comparison of soil physico-chemical properties, OUT distribution analysis and PCA analysis of saline pot planting soil with original saline soil and original cultivated soil with the application of DY1-3 bacterial suspension. (**a**) is the comparison of soil physico-chemical properties; (**b**) is the OUT distribution analysis; and (**c**) is the PCA analysis. ns indicates no significance; * indicates significant difference (*p* < 0.05); ** indicates highly significant difference (*p* < 0.01).

The results of the comparative analysis of the soil physicochemical factors of OY, YA, and OG are shown in Figure 16a. The results showed that the pH of both OY and OG was significantly higher than that of YA (*p* < 0.01), while the TS content in YA had a highly significant decrease (*p* < 0.01) from (0.58 ± 0.05) g/kg to (0.38 ± 0.02) g/kg compared with that of OY, and the AP content had a significant increase (*p* < 0.05) from (0.49 ± 0.08) g/kg to (1.30 ± 0.08) g/kg compared with that of OY. SOM content had a highly significant increase (*p* < 0.01) from (3.89 ± 0.30) g/kg to (4.50 ± 0.15) g/kg compared to OY, with a tendency to approach to TS, AP, and SOM content in OG.

Venn diagrams were used to analyse the composition of shared and endemic bacterial OTUs in OY, YA, and OG to visualise the similarity and overlap. The results showed that the total number of OTUs in YA and the number of OTUs shared with OG were higher than those in YB. A total of 5988 OTUs were found in the three soils, of which 2669 OTUs, or 44.57% of the total number, were detected in OY, and 2947 OTUs, or 49.22% of the total number, were detected in YA, and the number of OTUs shared by OY and OG was 258; the number of OTUs shared between OY and OG was 258; the number of OTUs shared between YA and OG was 485. It can be seen that the application of DY1-3 bacterial suspension and the planting of potted plants changed the colony structure of the original saline soil, which led to the change in the grouping of OTUs, and at the same time, the application of DY1-3 bacterial suspension and the planting of potted plants increased the number of OTUs shared between saline soil and ploughed soil and led to the change in the colony structure of the saline soil towards the direction of the ploughed soil.

The results of principal component analysis of bacterial communities in OY, YA, and OG are shown in Figure 16c. The differences and distances between samples were reflected by analysing the OTU (97% similarity) compositions of different samples; the more similar the sample compositions were, the closer they were to each other. PC1 and PC2 explained 61.98% and 18.25% of the differences between the samples, respectively, for a total of 80.23%. From the figure, it can be seen that the application of DY1-3 bacterial suspension and the planting of potted plants caused the microbial community composition in saline soil to differ, and the distribution of the samples changed from OY to YA, and the distribution of the samples of YA was closer to the distribution of the samples of OG on the axis of PC1, which had a relatively high amount of explanation. This indicates that the application of bacterial suspension and the planting of potted plants can make the microbial composition in saline soil have a tendency to be closer to the microbial composition in arable soil.

## 4. Discussion

### 4.1. Differences in Physico-Chemical Properties and Microbial Community Structure of Different Soil Types

Soil is one of the foundations for crop growth and development, and differences in soil physico-chemical properties and microbial community composition in different soils may result in different soil utilisation [36]. It was found that there were highly significant differences in several soil physico-chemical properties in GA and GB; AP, AK, TN, and SOM contents and pH were highly significant (*p* < 0.01) higher in OG than in OY, while TS content was highly significant (*p* < 0.01) higher in OY than in OG. And among the many soil physico-chemical properties mentioned above, TS is one of the most important environmental factors affecting plant growth and development and the composition and diversity of microbial communities. TS was similarly found to be the dominant factor affecting the composition of soil microbial community in RDA analysis and Spearman analysis. It has been shown that soil bacterial community diversity decreases with increasing salinity [37], so the difference in TS content between the two soils may be responsible for the higher bacterial community richness and diversity in OG than in OY. In the abundance analysis of bacterial community composition at the phylum level, Proteobacteria, Actinobacteriota, and Acidobacteria were found to be the main dominant phyla in both soil samples, which is consistent with the results of existing studies [36,38]. However, the relative abundance of Proteobacteria and Actinobacteria in OY was significantly higher than that in arable soils (*p* < 0.01), and the reason for this difference was mainly due to the difference in TS between the two types of soils. Kao et al. found that Proteobacteria showed a high salt ecotope preference [37], and Zenova et al. found that Actinobacteriota was salt tolerant [39], so its abundance would be higher in lower salt stress environments than in salt-free environments. 

The differences between the two types of soils in terms of physical and chemical properties, as well as the differences in the composition and diversity of microbial communities mainly caused by the differences in TS content, may lead to differences in microbial metabolic activities in the environment, and the microbial metabolic activities in the environment will indirectly affect the environment and then affect the plants in the soil, and these synergistic constraints determine the differences in agricultural use of soils. So it is of great significance to explore the soil physico-chemical properties and microbial community composition of soil in different types of soil for the rational use of soil resources and saline soil improvement.

### 4.2. Effects of DY1-3 Solution on the Growth of Maize Seedlings, Soil Physico-Chemical Properties and Bacterial Community Structure in Arable Soil

This part of the study revealed the effects of DY1-3 bacterial suspension application on the growth of maize plants, soil physico-chemical properties, and the bacterial community structure in arable soil, and compared the soil before and after potted planting in order to analyse the effects of potted planting on the soil physico-chemical properties and bacterial community structure in the arable soil.

It was found that potting treatment could change the composition of soil physico-chemical properties and bacterial community structure in arable soils, and that the DY1-3 strain could have a significant positive effect on the growth of maize seedlings. In terms of morphological indexes, the application of DY1-3 bacterial suspension highly significantly increased the plant height of maize seedlings (*p* < 0.01) and significantly reduced the number of root branches of maize seedlings (*p* < 0.05) compared with that of maize seedlings cultured by applying sterile water. In relation to the results of the physical and chemical properties of the original arable soil, it can be seen that the average pH of the original arable soil was 8.29, which was alkaline, and this alkaline soil environment might have induced the bacterial strains to produce certain biotrophic substances, such as IAA, so that the height of the maize plants increased significantly after the application of bacterial suspension, and the number of root branches was significantly reduced due to the reduction of the environmental pressure. In terms of physiological indices, the application of DY1-3 solution significantly increased the SOD content (*p* < 0.01) and total chlorophyll content (*p* < 0.05) of maize seedlings. The changes in these two indicators were likewise related to the IAA-producing and phosphorus-solubilising mechanisms of the DY1-3 strain applied to the inter-root soil of the plants. Li Wenxiang et al. similarly found that the application of the ACC deaminase-containing inter-root facultative bacterium F23 significantly increased the POD activity, CAT activity, chlorophyll a, and chlorophyll b in cut-flowering moonflower white lychee [40]. 

Soil provides a growing environment for plants, and its soil physico-chemical properties in turn directly affect plant growth and development [41]. Soil physico-chemical properties were measured before and after potting; it was found that the cultivation of maize plants altered the levels of AK and TS in arable soils and that the application of DY1-3 bacterial suspension increased the utilisation of SOM in the soil by maize plants. It was specifically shown that compared to AP and TN, maize utilised more AK in the soil during the growth process, and there was a highly significant (*p* < 0.01) decrease in AK content in GA and GB compared to OG, indicating that changes in the content of potash are one of the important constraints in the growth process of maize, which corroborates with the findings of Li Qiuyue et al. [42]. As for the RDA analysis of soil physicochemical factors and bacterial community composition, it was found that AK content in arable soils was likewise a key factor influencing the structural composition of bacterial communities. It has been shown that a certain range of low amounts of fast-acting potassium was positively correlated with microbial community abundance in the soil, while excessive amounts of potassium negatively affected the microbial community in the soil [43,44]. This indicates that in soils with high AK content, AK in the soil can be utilised through crop cultivation, thus reducing the constraints of AK on the richness of soil microbial communities and increasing the abundance of beneficial microorganisms in the soil to promote nutrient cycling in the soil, which in turn serves the purpose of soil improvement. And pot planting caused a highly significant increase in pH in arable soil, probably due to the utilisation of other nutrients and production of some acidic root secretions by the plant during growth. Meanwhile, the SOM content in GA was significantly lower than that in OG (*p* < 0.05), suggesting that the applied DY1-3 may have facilitated SOM utilisation by plants by converting SOM into carbon dioxide, water, and inorganic salts [42,45,46].

The inter-root soil is the most direct site for nutrient uptake by plants, and soil fertility is positively correlated with the total number of inter-root microorganisms, while changes in the composition of the soil microbial community are also an important factor affecting soil fertility and plant growth and health, and the inter-root bacteria, as a biofungal agent, can survive and proliferate in the inter-root of the plant and induce changes in the inter-root microbial community of the plant [47,48]. Further analysis of the structure and composition of the bacterial community in the soil before and after potting showed that the application of DY1-3 bacterial solution and potting could change the structure of the bacterial community in the arable land and increase the diversity and abundance of the bacterial community in the arable land. It was specifically shown that the number of total and exclusive OTUs was higher in GA than in OG and GB, while the cultivation of potted plants significantly increased both the Shannon and Simpson indices in arable soils, and the Simpson index was significantly higher in GA compared to GB (*p* < 0.05), and the PCA results similarly showed that the sample distribution areas of OG, GA, and GB appeared clearly differentiated. The results indicated that the application of DY1-3 bacterial suspension and the planting of pot plants could change the structure of bacterial communities in arable soil and increase the diversity and richness of the bacterial communities in arable land. Lv Guirong and He Minghuang also found that the application of microbial fertiliser could significantly change the bacterial community structure and increase the richness of bacterial community in soil [49,50], which is in line with the results of our study. The relative abundance of different bacterial phyla and genera in OG, GA, and GB was further analysed. At the phylum level, the relative abundance of Proteobacteria and Acidobacteria in GA and GB increased significantly (*p* < 0.05) compared to that of OG. At the genus level, *Vicinamibacteraceae* and *RB41* showed highly significant (*p* < 0.01) decreases in relative abundance in both GA and GB compared to their abundance in OG. Among them, Proteobacteria contain a large number of functional groups of oligotrophic microorganisms, and a considerable number of taxa have nitrogen-fixing capacity and can utilise nutrients produced by the decomposition of various organic materials for metabolic activities [51], whereas the Acidobacteria phylum can drive the chemical cycling of carbon and nitrogen elements in organisms and the environment and is suitable for low-carbon soils [38], and the changes in their abundance were associated with the previously mentioned soil after potting. The reduction of organic matter content in the soil after potting may be related.

The above results suggest that the application of DY1-3 bacterial suspension did not have much effect on the physico-chemical factors and soil bacterial communities we measured under non-stressful conditions but more on the plant-promoting aspects. This may be related to the fact that our bacteria come from stressful environments, where they are better able to perform their ameliorative effects on the soil, whereas in more suitable soil environments they may be able to produce and metabolise more pro-biotic substances to help plant growth. Whereas plants can influence the soil flora through root secretions and have a strong influence on soil physical and chemical factors (uptake of physical and chemical elements for growth), the microbial community in the soil may change in abundance due to the mediation of certain soil elements, but not in general species. In a stress-free environment, microorganisms may be more likely to produce plant-friendly metabolites for better plant growth and development than for environmental amelioration, but elemental changes in the soil may still affect the abundance of the microbial community.

### 4.3. Effects of the Application of DY1-3 Bacterial Suspension on the Growth of Maize Seedlings, Soil Physico-Chemical Properties, and Bacterial Community Structure in Saline Soil

This part of the study revealed the effects of DY1-3 bacterial suspension on the growth of maize plants, the soil physico-chemical properties of saline soil, and the bacterial community structure in saline soil, and compared the soil before and after potting in order to analyse the effects of potting on the soil physico-chemical properties and bacterial community structure in saline soil.

Relevant studies have found that when maize is subjected to salt stress, crop growth and development will be inhibited, mainly in terms of growth slowdown, root elongation, and root area reduction, etc., and the PGPB can alleviate the crop stress by promoting the accumulation of proline and sugars in the crop seedlings, thus reducing the osmotic potential of the crop [52,53]. In this study, it was found that in terms of morphological indicators, the application of DY1-3 bacterial suspension significantly increased the plant height of maize seedlings (*p* < 0.05) and highly significantly reduced the root length and the number of root branches (*p* < 0.01) compared to maize seedlings cultured with the application of sterile water. This suggests that DY1-3, as an inter-root biotrophic bacterium, may promote the accumulation of sugars in maize seedlings through the production of certain biotrophic substances such as EPS and IAA, etc. At the same time, DY1-3 may also alleviate environmental stresses on maize seedlings through the production of ACC deaminase, which reduces the toxicity of over-synthesised ethylene under salt stress, resulting in a decrease in the length of the primary root and the number of root branches and an increase in the height of the maize seedlings, which is in line with previous research results in the laboratory [31]. In terms of physiological indices, the application of DY1-3 bacterial suspension highly significantly increased (*p* < 0.01) SOD, POD, CAT, and chlorophyll contents and highly significantly decreased (*p* < 0.01) MDA contents in maize seedlings. Similarly suggesting that DY1-3 can alleviate environmental stresses on plants under saline stress by producing certain pro-biotic substances. Singh et al. similarly found that inoculation of ACC deaminase-producing pro-biotic bacteria alleviated adversity stresses on wheat by increasing photosynthetic rate and decreasing MDA content [54]. Soil physico-chemical properties, such as fast-acting nutrients, organic matter content, and pH, are important indicators of soil substrate availability and its associated health status [55]. 

Soil physico-chemical properties were measured before and after pot planting, and it was found that pot planting resulted in highly significant decreases in pH and TS content, highly significant increases in AP, TN, and SOM content in saline soils (*p* < 0.01), and significant decreases in TS and AP content in YA compared to YB (*p* < 0.05), suggesting that planting in pots and the application of DY1-3 bacterial suspension play an ameliorating effect on some nutrient contents of saline soils. Since the planting of certain saline-tolerant crops can improve soil salinisation, reduce the salt content of soil, and increase the content of effective elements in soil [56], the TS content of soil was significantly reduced and the content of AP, TN, and SOM was significantly increased after pot planting. The study of Xing Jiaming et al. also proved that crop planting plays a significant role in reducing the salt content of salinised soil layers and increasing soil organic matter [57]. The study of He Guoxing et al. likewise proved that the planting of salinity-tolerant plant alfalfa accelerated the transformation and utilisation of soil nutrients [58]. Combined with the previous section, the differences in TS content in the potting soil of different treatment groups may be due to the changes in root secretion content caused by the response mechanism of the DY1-3 strain under salt stress on the growth and development of maize plants, which indirectly affected the soil salinity content, and the changes in AP content may be due to the fact that the DY1-3 strain promoted the growth of maize plants through the production of some biomass and accelerated the plant response to phosphorus. 

Microorganisms are commonly found in soil ecosystems, and understanding the structure of soil microbial communities and their roles in salinised soils can help to understand the regulatory process of salinised soil improvement [59]. Bacterial community diversity and structural composition were analysed in potting soil and virgin saline soil from two different treatment groups. It was found that the total OTUs, exclusive OTUs, Shannon index, and Chao1 index were higher in YA than in OY and YB (*p* < 0.05), and there was also a significant increase in the Simpson index and Chao1 index in YB compared to OY (*p* < 0.05). The PCA results similarly showed that the cultivation of potted plants and the application of the DY1-3 bacterial suspension resulted in a significant increase in YA, YB, and OY sample distribution areas that appeared to be significantly differentiated. It indicated that the application of DY1-3 bacterial suspension could increase the richness and diversity of bacterial communities in saline soil, while the planting of maize plants could only increase the diversity of bacterial communities in saline soil but did not significantly enhance the richness of the bacterial communities [60,61]. The relative abundance of different phyla and genera of bacteria in OY, YA, and YB was further analysed. It was found that Proteobacteria and Actinobacteria were absolutely dominant in YB and OY [62]. The relative abundance of Proteobacteria in YB was significantly higher than that in OY, indicating that the cultivation of maize plants may improve saline soil by increasing the relative fraction of Proteobacteria in saline soil to improve the nutrient cycling in the soil in the key aspects of nitrogen, sulphur, and phosphorus cycles [63], whereas the dominant phylum in YA was changed from Cyanobacteria to Acidobacteriota. Both Cyanobacteria and Acidobacteriota are soil-beneficial bacteria with the ability to promote soil nutrient cycling [64], and it is speculated that the change between the two in OY and YA may be related to antagonistic and synergistic effects between the strains [10].

The above results showed that the soil flora changed under saline environment but the change in the flora did not significantly increase the content of soil physicochemical factors, while the plant stress indicators were greatly improved. This suggests that the bacterial flora will preferentially help the plant to resist stress and help the plant to take up some essential elements to assist the plant growth under the stressful environment.

### 4.4. Amelioration Effect of DY1-3 Bacterial Suspension Application and Planting of Maize Seedlings on Saline Soil

Soil is one of the basic substances for crop growth and development, and changes in its fertility have a great impact on the quality and yield of crops [65]. Soil physico-chemical properties are important indicators of soil fertility, such as soil pH, which is an important indicator reflecting the level of soil acidity and alkalinity, and the change in pH in the soil affects the activity of microorganisms, decomposition of minerals and organic matter, etc., in the soil [60,66,67]. For example, nitrogen, phosphorus, potassium, etc. are essential mass nutrients for crop growth, and the level of their content directly reflects the soil fertility. In agricultural production, chemical fertilisers are often applied to provide sufficient nitrogen, phosphorus, potassium, and other elements for plant growth, but the excessive and irrational application of chemical fertilisers caused by soil compaction and soil fertility decline has become an important factor restricting agricultural production [67]. Numerous studies have found that microbial fertilisers can improve soil fertility in a green way, and the cultivation of some saline-tolerant crops such as alfalfa, liquorice, and maize can also improve saline soils [68,69,70]. In this study, it was found that the effect of the cultivation of maize plants and the application of DY1-3 bacterial suspension on soil nutrients was analysed by comparing the differences in soil physico-chemical properties between OY, YA, and OG by potting the saline soils and applying DY1-3 bacterial suspension. It was found that pH and TS contents in YA were highly significantly decreased (*p* < 0.01) and AP and SOM contents were significantly and highly significantly increased (*p* < 0.05) compared to OY, and the contents of TS, AP, TN, and SOM in YA had a tendency to be closer to those in OG. Microorganisms are the main participants and contributors to energy transport and nutrient cycling in soil ecosystems and one of the important indicators of soil health [71]. It has been shown that the complexity and diversity of soil microorganisms is one of the important features for ecosystem stability and maintenance of healthy plant growth, nutrition, and yield, among others [72]. For example, Arthrobacter had an enhancing effect on enzyme activities such as soil urease and catalase, and Gemmatimonas effectively increased the abundance of beneficial bacteria in soil and inhibited the growth of pathogenic bacteria [73,74]. In this study, it was found that the application of DY1-3 bacterial suspension and the planting of potted plants could cause the bacterial community composition in saline soil to show a tendency to approach the bacterial community composition in arable soil. It was shown that the total OTUs, Shannon index, Simpson index, and Chao1 index in YA increased (*p* < 0.05) compared with OY, and the application of DY1-3 bacterial suspension increased the OTUs shared by saline soil and arable soil from 258 to 485; meanwhile, the elevated Simpson index arrived at the same level as that of the Simpson index in arable soil. The PCA results likewise showed that the sample distribution of YA was close to that of OG on the PC1 (61.98%) axis, which had a relatively high amount of explanation. The relative abundance of different phyla and genera in OY, YA, and OG was further analysed. At the phylum level, the relative abundance of the phyla Acidobacteriota, Gemmatimonadota, and Chloroflexi in YA increased, and the relative abundance of the phylum Myxococcota decreased, showing a tendency to move closer to the distribution of bacterial flora in arable soils. At the genus level, the relative abundance of genera such as *Pseudomonas* and *Chloroplast* decreased in YA, while the relative abundance of genera such as *Sphingomonas* and *Vicinamibacteraceae* increased, also showing a tendency to move closer to the distribution of bacterial communities in arable soils. The results of the above analyses of soil physico-chemical properties and bacterial community composition showed that the application of bacterial suspension and the planting of potted plants could make the contents of TS, AP, TN, and SOM, as well as the bacterial community composition of saline soils, closer to that of arable soils, which indicated that the application of bacterial suspension and the planting of potted plants had a certain positive effect on the improvement of saline soils and the enhancement of their fertility.

## 5. Conclusions

Soil salinity has become an important constraint to agricultural production, and soil improvement using microbial agents has become a key measure to improve soil fertility and increase crop yields. In this study, the use of microbial agents in both arable and saline soils, which have obvious differences in physical and chemical properties and bacterial community composition, was able to promote the growth of maize seedlings and change the physical and chemical properties and bacterial community structure of both types of soils, and achieve soil improvement and soil fertility in saline soils, from which we generate the following conclusions:

1. There were significant differences between arable and saline soils in terms of soil physico-chemical properties and bacterial community structure composition, while TS was the main environmental factor influencing the composition and diversity of microbial communities. Differences in microbial metabolism between the two soils due to differences in various soil physico-chemical properties and differences in microbial community composition and diversity, mainly due to differences in TS content, may be a key determinant of differences in agricultural use of the soils.
2. In arable soils, the application of DY1-3 bacterial suspension did not have much effect on the physicochemical factors and soil bacterial communities we measured, but more on the pro-biotic aspect of the plants, which could increase the plant height, SOD, and chlorophyll content of maize seedlings, but the elemental variations in the soil could still have an effect on the abundance of microbial communities. This may be related to the fact that our bacteria come from stressful environments, in which they are better able to carry out soil amelioration effects, whereas they may produce and metabolise more pro-biotic biomass to help plant growth in more suitable soil environments. Moreover, maize utilised more AK in soil during growth, while in the RDA analysis of soil physicochemical factors and bacterial community composition, it was found that AK content was also a key factor influencing the structural composition of the bacterial community.
3. In saline soil, the application of DY1-3 bacterial suspension also had a significant positive effect on the growth of maize plants and the richness of bacterial community, as well as an obvious alleviation effect on the environmental stresses suffered by maize plants during the growth process. It increased the plant height, SOD, POD, CAT, and chlorophyll content and reduced the root length, root branch number, and MDA content of maize seedlings, which could lead to an increase in the diversity and abundance of the bacterial community in saline soil. And the application of DY1-3 bacterial suspension and the planting of potted plants can make the TS in saline soil decrease significantly, playing a role saline soil improvement.
4. The application of DY1-3 bacterial suspension and the planting of maize seedlings had a certain positive effect on the improvement of saline soil and the improvement of fertility, which made the content of TS, AP, TN, and SOM, as well as the composition of the bacterial community in saline soil, appear close to that of arable soil.

## Figures and Tables

**Table 1:** Statistical table of valid data.

Sample Name	Raw Reads	Clean Reads	Effective%	OTUs
OG1	74,173	72,497	97.74	2755
OG2	55,163	53,284	96.59	2831
OG3	65,880	64,168	97.40	2795
OG4	70,811	68,672	96.98	2996
OG5	72,199	69,562	96.35	2909
OY1	35,280	34,935	99.02	1734
OY2	56,820	56,307	99.10	1822
OY3	34,110	33,903	99.39	1538
OY4	27,619	27,431	99.32	1464
OY5	38,195	37,911	99.26	1456

Raw Reads are base sequences after quality control splicing; Clean Reads are base sequences that should be used for subsequent analysis after further removal of chimeras and short sequences; Effective% is the percentage of Clean Reads versus Raw Reads; OTUs are the final number of OUTs obtained from sequencing.

**Table 2:** Statistical results of diversity indices of different types of soil bacterial communities.

Sample	OTUs	Shannon	Simpson	Chao1	Goods Coverage
OG	2857 ± 96 a	9.7569 ± 0.0960 a	0.9967 ± 0.0002 a	3691.515 ± 155.813 a	0.9652 ± 0.0018 a
OY	1602 ± 166 b	7.4808 ± 0.5097 b	0.9563 ± 0.0197 b	1866.832 ± 216.090 b	0.9861 ± 0.0027 b

Different lower case letters in the same column indicate a significant level of difference (*p* < 0.05).

**Table 3:** Statistical table of valid data.

Sample Name	Raw Reads	Clean Reads	Effective%	OTUs
GA1	63,783	60,552	94.93	3871
GA2	70,853	68,170	96.21	4107
GA3	74,169	70,836	95.51	4274
GA4	77,721	72,862	93.75	3867
GB1	77,223	74,558	96.55	3956
GB2	74,538	71,714	96.21	4022
GB3	71,954	69,852	97.08	3662
GB4	76,699	73,792	96.21	4020

Raw Reads are base sequences after quality control splicing; Clean Reads are base sequences that should be used for subsequent analysis after further removal of chimeras and short sequences. Effective% is the percentage of Clean Reads versus Raw Reads; OTUs are the final number of OUTs obtained from sequencing.

**Table 4:** Statistical results of soil bacterial community diversity indices of the original ploughed soil and the ploughed soil after potted plants.

Sample	OTUs	Shannon	Simpson	Chao1	Goods Coverage
GA	4029 ± 198 a	10.1499 ± 0.1251 a	0.9977 ± 0.0002 a	4870.657 ± 284.790 a	0.9783 ± 0.0016 a
GB	3915 ± 171 a	10.0085 ± 0.1465 a	0.9973 ± 0.0003 b	4864.216 ± 230.542 a	0.9777 ± 0.0011 a
OG	3880 ± 125 a	9.9436 ± 0.1012 b	0.9970 ± 0.0002 c	4681.391 ± 131.868 a	0.9789 ± 0.0009 a

Different lower case letters in the same column indicate a significant level of difference (*p* < 0.05).

**Table 5:** Statistical table of valid data.

Sample Name	Raw Reads	Clean Reads	Effective%	OTUs
YA1	32,519	31,988	98.37	1894
YA2	45,575	44,645	97.96	1927
YA3	39,510	38,830	98.28	1841
YA4	40,389	39,622	98.10	1772
YB1	33,552	32,136	95.78	1543
YB2	47,322	45,222	95.56	1588
YB3	64,320	60,962	94.78	1709
YB4	72,840	70,647	96.99	1615

Raw Reads are base sequences after quality control splicing; Clean Reads are base sequences that should be used for subsequent analysis after further removal of chimeras and short sequences; Effective% is the percentage of Clean Reads versus Raw Reads; OTUs are the final number of OUTs obtained from sequencing.

**Table 6:** Statistical results of bacterial community diversity index of original saline soil and post-potting saline soil.

Sample	OTUs	Shannon	Simpson	Chao1	Goods Coverage
YA	1858 ± 68 a	8.7032 ± 0.1808 a	0.9895 ± 0.0039 a	2120.417 ± 66.787 a	0.9851 ± 0.0007 b
YB	1613 ± 70 b	7.9511 ± 0.2400 b	0.9812 ± 0.0052 a	1894.056 ± 103.166 b	0.9858 ± 0.0010 b
OY	1578 ± 156 b	7.5832 ± 0.5175 b	0.9613 ± 0.0200 b	1821.878 ± 190.620 c	0.9870 ± 0.0020 a

Different lower case letters in the same column indicate a significant level of difference (*p* < 0.05).

## Data Availability

The data presented in this study are available upon request from the corresponding authors.

## References

[1] Paulo P., Damià B., Panos P. (2020). Soil and water threats in a changing environment. Environ. Res..

[2] Li X., Yang J., Xu X., Yang J., Zheng G., Xiao H. (2010). Research Advances on Alkaline Soil Improvement by Flue Gas Desulphurization Byproducts. Soils.

[3] Daliakopoulos I.N., Tsanis I.K., Koutroulis A., Kourgialas N.N., Varouchakis A.E., Karatzas G.P., Ritsema C.J. (2016). The threat of soil salinity: A European scale review. Sci. Total Environ..

[4] Yanmin Y., Licheng W., Hongtao W., Zhenhua X., Haiying L., Chunxu L., Zhongyi S., Ping Y. (2024). Effects of Saline-Alkali Stress on Growth Characteristics and Yield of Rice. Heilongjiang Agric. Sci..

[5] Ondrasek G., Rengel Z. (2021). Environmental salinization processes: Detection, implications & solutions. Sci. Total Environ..

[6] Chendi S. (2020). Long-Term Positioning Study on the Effects of Different Treatments of Reed Straw on Improving Saline-Alkali Land. Int. J. Ecol..

[7] Li S., Chen P., Liu H., Hao J., Zhou Y., Shi L. (2019). Mechanism and ecological effects of arbuscular mycorrhizal fungi on improving salt tolerance of plants in coastal saline–alkaline land. Ecol. Environ. Sci..

[8] Wang X.F., Yang S. (2020). An Overview of Saline-alkali Land Improvement Measures. Constr. Technol..

[9] Yin X.L., Cheng C., Ding G.D., Zhang X.M., Gao G.L., Bao S.Z. (2020). Improvement and effects of calcium-containing wastes on saline-alkali soil and growth of Lespedeza bicolor Turcz. Sci. Soil Water Conserv..

[10] Liu C., Wan C.C., Song X., Xia G.F., Ao N., Wang K.M., Wang J. (2024). Effects of effective microorganisms on growth promotion and the rhizosphere eukaryotic community structure of pepper in Xinjiang, China. Chin. J. Appl. Ecol..

[11] Feng Y., Yang D., Zhao F., Hou X., Li Q., Zeng L., Lu H., Wang B. (2024). Effects of Different Microbial Agents on the Growth of Foxtail Millet and Enzyme Activities of Soil. Heilongjiang Agric. Sci..

[12] László K., Rita B., Dóra B., Henrietta A., Orsolya K., Gordana R., András V., Viktor D., Csaba V. (2024). Recent advances in the use of Trichoderma-containing multicomponent microbial inoculants for pathogen control and plant growth promotion. World J. Microbiol. Biotechnol..

[13] Li J., Wen Y., Fang Z., Yang W., Song X. (2024). Application of cold-adapted microbial agents in soil contaminate remediation: Biodegradation mechanisms, case studies, and safety assessments. RSC Adv..

[14] Yao D., Niu S.Q., Zhao Q., Cao J., Han Q., Li H., Gou J., Zhang J. (2020). Induced salt tolerance of ryegrass by Bacillus subtilis strain WM13-24 from the rhizosphere of Haloxylon ammodendron. Acta Ecol. Sin..

[15] Jordan E., Guilhem E., Marie-Lara E., Bruno T., Yvan M., Laurent L., Florence W., Claire P. (2013). Plant growth-promoting rhizobacteria and root system functioning. Front. Plant Sci..

[16] Rawat P., Das S., Shankhdhar D. (2020). Phosphate-Solubilizing Microorganisms: Mechanism and Their Role in Phosphate Solubilization and Uptake. J. Soil Sci. Plant Nutr..

[17] Jiawei W., Aoping T., Jianli L., Wanpeng T., Jiamin Y., Miao G., Biwen S., Zhirou S. (2024). Screening of rhizosphere growth\|promoting bacteria of desert plants and its promoting effects on silage maize seedlings. Agric. Res. Arid Areas.

[18] Abdelkefi N., Louati I., Mechichi H.Z., Sayahi N., El-Sayed W., Nayal A., Ismail W., Hanin M., Mechichi T. (2024). Enhanced salt stress tolerance in tomato plants following inoculation with newly isolated plant growth-promoting rhizobacteria. Sci. Hortic..

[19] Chamkhi I., El Omari N., Balahbib A., Menyiy N., Benali T., Ghoulam C. (2022). Is—the rhizosphere a source of applicable multi-beneficial microorganisms for plant enhancement?. Saudi J. Biol. Sci..

[20] Zhang X., Yang Z., Wang L., Yue Y., Wang L., Yang X. (2023). The effects of plant growth-promoting rhizobacteria on plants under temperature stress:A meta-analysis. Rhizosphere.

[21] Wu Y., Xing S., Zhang X., Guan F., Wu H., Zai D., Li C., Xu L., Li X., Zou Y. (2023). Studies on the application of inter-root biotrophic bacteria to salt stress in plants. Agric. Technol..

[22] Wu Z., Song J., Zhu S., Zhao X., Yang X., Ren J., Chen F. (2023). Effects of Plant Growth-Promoting Microorganisms on Rhizosphere Microbial Community and the Leaf Pigment Composition of Liquidambar formosana. Sci. Silvae Sin..

[23] Yuan B., Tian C., Hu Y. (2023). Effects of Biochar and Plant Growth Promting Rhizobacteria on soil physico-chemical properties and Microbial Comunity Composition of Salinized Soil. Shandong Agric. Sci..

[24] Guo H., Wang F., Tian L., Wang C., Li W., Fang B., Li X., Wang Z. (2024). Bacterial diversity and influencing factors in soil sediments of Yuncheng Salt Lake, Shanxi. Acta Microbiol. Sin..

[25] Li M., Bi J., Wang J. (2020). Bacterial community structure and key influence factors in saline soil of different sites in Ningxia. Acta Ecol. Sin..

[26] He H., Chen J., Wang T., Tang M., Liang J., Cao Z., Xiao M., He M., Li Y., Li X. (2024). Influence of soil physicochemical properties on microbial diversity in different production areas of Chinese Cordyceps in Qinghai province. Southwest China J. Agric. Sci..

[27] Ma L., Gao W., Mi H., Tang J., Li M., Huang S. (2021). Soil microbial community characteristics in greenhouse vegetable production under different fertilization patterns based on metagenomic analysis. Plant Nutr. Fertil. Sci..

[28] Zhang M., Wang E., Zhang Y., Liu J., Xu L., Chen Y. (2024). Isolation and characterization of Bacillus subtilis S1 capable of inducing resistance against powdery mildew and promoting cucumber growth from cucumber rhizosphere. Microbiol. China.

[29] Hou Y., Wei C., Zeng W., Hou M., Wang Z., Xu G., Huang J. (2024). Application of rhizobacteria to improve microbial community structure and maize (*Zea mays* L.) growth in saline soil. Environ. Sci. Pollut. Res. Int..

[30] Shi Y., Yuan Y., Feng Y., Zhang Y., Fan Y. (2023). Bacterial Diversity Analysis and Screening for ACC Deaminase-Producing Strains in Moss-Covered Soil at Different Altitudes in Tianshan Mountains—A Case Study of Glacier No. 1. Microorganisms.

[31] Yuan Y., Shi Y., Liu Z., Fan Y., Liu M., Ningjing M., Li Y. (2023). Promotional Properties of ACC Deaminase-Producing Bacterial Strain DY1-3 and Its Enhancement of Maize Resistance to Salt and Drought Stresses. Microorganisms.

[32] Sun D., Liu X., Yang J., Yu J., Wang Z., Zhou D., Yu Y., Ning K. (2023). Effects of returning years from farmland to wetland on the content and distribution of soil iron oxides in the Yellow River Delta. Chin. J. Ecol..

[33] Bao S. (2000). Soil and Agricultural Chemistry Analysis.

[34] Caporaso J.G., Lauber C.L., Walters W.A., Berg-Lyons D., Lozupone C.A., Turnbaugh P.J., Fierer N., Knight R. (2011). Global patterns of 16S rRNA diversity at a depth of millions of sequences per sample. Proc. Natl. Acad. Sci. USA.

[35] Zhao L., Xu Y., Chang J., Li M., Zhang Y., Dang Y., Wang M., Cheng Y., Zhang B. (2016). Screening, resistance and growth-promoting effect of endophytic bacteria with ACC deaminase activity isolated from soybean nodules. Acta Microbiol. Sin..

[36] Tianyun S., Jianjing Z., Anhong L. (2020). Effects of soil physicochemical properties on microbial communities in different ecological niches in coastal area. Appl. Soil Ecol..

[37] Zhang K., Shi Y., Cui X., Yue P., Li K., Liu X., Tripathi B.M., Chu H. (2019). Salinity Is a Key Determinant for Soil Microbial Communities in a Desert Ecosystem. mSystems.

[38] Hu F., Wang F., Han X., Xu M., Fu Y., Yan J., Jia Z., James M., Jiang X. (2022). Succession of Microbial Community in Typical Black Soil under Different Land Use Pattern. Acta Pedol. Sin..

[39] Zvyagintsev D.G., Zenova G.M., Oborotov G.V. (2009). Moderately haloalkaliphilic actinomycetes in salt-affected soils. Eurasian Soil Sci..

[40] Li W., Zhang T., Li Y., Li X., Huang H., Huang M. (2023). Effect of Rhizobacteria Containing ACC Deaminase on Growth of Rose Bush. Fujian J. Agric. Sci..

[41] Zhang H., Zhang S., Zhang Y., Zhang T. (2019). Genetic 16S rRNA Diversity of Soil Microbes in Rhizosphere of Chinese Yam and Its Influencing Factors. Acta Pedol. Sin..

[42] Li C., Wei X., Zhao Z. (2017). Effects of different fertiliser ratios on the growth and nitrogen, phosphorus and potassium content of maize stover. J. Zhejiang Agric. Sci..

[43] Pei Y., Ren S., Li C., Chen X., Xia T., Jiang M., Yin M. (2022). Study on arbuscular mycorrhizal fungi difference in roots and rhizosphere soil in healthy and black shank infected tobacco Yunyan 121. J. South. Agric..

[44] Yang S., Deng L., Huang B., Wang Y., Huang Y., Xiao W., Zheng X., Su Y., Ren Z., Yin M. (2023). Correlation analysis between diversity of arbuscular mycorrhi-zal fungi in roots and soil physicochemical properties of *Panax notoginseng* (Burk.)F.H.Chen at different ages. J. South. Agric..

[45] Wang J., Wang H., Zhao X., Zhao L. (2020). Effect of Straw Addition on Acidity and Buffering Performance of Soil with Different Organic Contents. J. Soil Water Conserv..

[46] Wu L., Lin X., Lin W. (2014). Advances and perspective in research on plant-soil-microbe interactions mediated by root exudates. Chin. J. Plant Ecol..

[47] Shi Y., Shi D., Wang Y., Wu F., Zhou X. (2022). Effects of Applying Different Rhizosphere Growth-promoting Bacteria on Pepper Growth and Soil Microbial Community. China Veg..

[48] Elizabeth T., Dwipendra T., Mohan Chandra K., Chandradev K.S., Narayan C.T. (2017). Root colonization by host-specific rhizobacteria alters indigenous root endophyte and rhizosphere soil bacterial communities and promotes the growth of mandarin orange. Eur. J. Soil Biol..

[49] He M., Xi W., Wang D., Pan H., Zhu X., Liu Z. (2023). Effect of Microbial Fertilizer on Heavy Metal Content, Microbial Community Structure and Function in Aquacultural Sediment. Saf. Environ. Eng..

[50] Lv G. (2023). Effect of microbial fertiliser on soybean yield and soil microbial diversity. China Agric. Technol. Ext..

[51] Liu J., Sui Y., Yu Z., Shi Y., Chu H., Jin J., Liu X., Wang G. (2014). High throughput sequencing analysis of biogeographical distribution of bacterial communities in the black soils of northeast China. Soil Biol. Biochem..

[52] Tang G., Li A., Zhou W., Wu T., Hou S., Hou Y., Zeng W. (2023). Effects of Rhizosphere Plant Growth Promoting Bacteria on Maize Growth Under Salt Stress. Water Sav. Irrig..

[53] Zhao W. (2019). Research Progress of Soil Salinization Stress on Maize. Heilongjiang Agric. Sci..

[54] Suman S., Arpita T., Chandan Singh C., Deepti B., Pooja S., Vikas K.P., Poornima V. (2020). Cold stress alleviation using individual and combined inoculation of ACC deaminase producing microbes in Ocimum sanctum. Environ. Sustain..

[55] Zhao W., Huang L. (2022). Stoichiometric characteristics and influencing factors of soil nutrients under different land use types in an alpine mountain region. Acta Ecol. Sin..

[56] Liu G., Gao Y., Shao Z., Dei C. (2024). Research Progress of Soil Salinization-Alkalization Remediation Technology. Heilongjiang Agric. Sci..

[57] Xing J., Yu H. (2024). Effects of planting alfalfa on improving saline-alkali soil in Huanghua City, Hebei Province. Anim. Breed. Feed.

[58] He G., Song J., Wen Y., Liu C., Qi J. (2020). Effects of different rhizobium fertilizers on alfalfa productivity and soil fertility. Acta Pratacult. Sin..

[59] Zhao W., Guo Q., Li S., Lu X., Dong L., Wang P., Zhang X., Su Z., Ma P. (2022). Application of Bacillus subtilis NCD-2 can suppress cotton verticillium wilt and its effect on abundant and rare microbial communities in rhizosphere. Biol. Control.

[60] Wang Y., Liu S., Li X., Wang S., Liu Q., Yin K., Zhang X. (2022). Effects of three saline-alkali tolerant growth-promoting bacteria on the rhizosphere microecology of mung bean. Agric. Res. Arid Areas.

[61] Zhao B., Pan F., Wang W., Meng C., Zhang X., Liu Z., Liu H., Han X. (2018). Effects of Biological Agent on Promoting Mahaleb Growth and Rhizospheric Bacterial Community. J. Shenyang Agric. Univ..

[62] Chou D., Liao Z., Xing Q., Wang H., Liu J., Zhao B. (2022). The correlation analyses between bacterial community and the crucial environmental factors in saline-alkali soil of Songnen Plain. Microbiol. China.

[63] Wu X., Wang R., Gao C., Gao S., Du L., Khan A., Barmon M., Guo S. (2021). Variations of soil properties effect on microbial community structure and functional structure under land uses. Acta Ecol. Sin..

[64] Ma Q., Yu J., Wei Y., Wang Y., Li Q., Teri G.L., Xia J., Lu Y., Xini N.G. (2020). Spatial differentiation of Cyanobacterial communities and their relationship with environmental factors in Xilin River basin. Acta Sci. Circumstantiae.

[65] Yang Y., Yin Y., Du W. (2024). Effects of planting techniques for two-season forage grass per year on soil enzyme activities in the alpine pasturing area of Northwesten Sichuan in Qinghai-Tibet Plateau. Grassl. Turf.

[66] Navidi M., Sheidai-Karkaj E., Plaza-Alvarez P.A., Xu X., Sasanifar S., Nazarnejad H., Ortega R., Lucas-Borja M.E., Zema D.A. (2024). Effects of banqueting on water infiltration and physico-chemical properties of soil in semi-arid lands. J. Arid Environ..

[67] Wang D., Niu S., Xu H., Zhao W., Yang X., Li W., Ma W., Sun Z. (2021). Status of soil fertility, nutrient balance, and environmental risk assessment in yam produc-tion of North China Plain. Chin. J. Appl. Ecol..

[68] Du Y., Zhang X., Wang Y., Fan B. (2023). Effects of Alfalfa Cultivation on Physiochemical Properties of Farmland Soil. Anim. Husb. Feed Sci..

[69] Li H. (2024). Microbial Fertilizers and Soil Conditioners Improvement Effect of Combined Application on Secondary Salinization Soil. North. Hortic..

[70] Yang X., Zhang K., Chang T., Shaghaleh H., Qi Z., Zhang J., Ye H., Hamoud A. (2024). Interactive Effects of Microbial Fertilizer and Soil Salinity on the Hydraulic Properties of Salt-Affected Soil. Plants.

[71] Hartmann M., Six J. (2022). Soil structure and microbiome functions in agroecosystems. Nat. Rev. Earth Environ..

[72] Edgar R.C. (2016). UNOISE2: Improved error-correction for Illumina 16S and ITS amplicon sequencing. bioRxiv.

[73] Zhang D., Ren L., Du H., Liu Y., Jin X., Chen J., Fang W., Yan D., Li Y., Wang Q. (2023). Treatment with microbial agents increases the relative abundance of beneficial microorganisms in the rhizosphere soil of ginger. Plant Prot..

[74] Jiang Y., Song Y., Jiang C., Li X., Liu T., Wang J., Chen C., Gao J. (2022). Identification and Characterization of Arthrobacter nicotinovorans JI39, a Novel Plant Growth-Promoting Rhizobacteria Strain from Panax ginseng. Front. Plant Sci..

